# Nociceptin/Orphanin-FQ Inhibits Gonadotropin-Releasing Hormone Neurons via G-Protein-Gated Inwardly Rectifying Potassium Channels

**DOI:** 10.1523/ENEURO.0161-18.2018

**Published:** 2018-12-26

**Authors:** Stephanie Constantin, Susan Wray

**Affiliations:** Cellular and Developmental Neurobiology Section, National Institute of Neurological Disorders and Stroke/National Institutes of Health, Bethesda, Maryland 20892-3703

**Keywords:** GIRK, GnRH, nociceptin, orphanin FQ, POMC

## Abstract

The pulsatile release of gonadotropin-releasing hormone (GnRH) is a key feature of the hypothalamic–pituitary–gonadal axis. Kisspeptin neurons in the arcuate nucleus (ARC) trigger GnRH neuronal activity, but how GnRH neurons return to baseline electrical activity is unknown. Nociceptin/orphanin-FQ (OFQ) is an inhibitory neuromodulator. ARC proopiomelanocortin (POMC) neurons, known to receive inputs from ARC kisspeptin neurons, contact GnRH neurons and coexpress OFQ in the rat. In the present study, the effect of OFQ(1-13) on GnRH neurons was determined in the mouse. We identified transcripts for the OFQ receptor [opioid receptor like 1 (ORL1)] in GnRH neurons, and, using two-model systems (explants and slices), we found that OFQ exerted a potent inhibition on GnRH neurons, with or without excitatory inputs. We confirmed that the inhibition was mediated by ORL1 via G_i/o_-protein coupling. The inhibition, occurring independently of levels of intracellular cyclic adenosine monophosphate, was sensitive to inwardly rectifying potassium channels. The only specific blocker of G_i/o_-protein-coupled inwardly rectifying potassium (GIRK) channels, tertiapin-Q (TPNQ), was ineffective in the inhibition of OFQ. Two GIRK activators, one sharing the binding site of TPNQ and one active only on GIRK1-containing GIRK channels, failed to trigger an inhibition. In contrast, protein kinase C phosphorylation activation, known to inhibit GIRK2-mediated currents, prevented the OFQ inhibition. These results indicate a specific combination of GIRK subunits, GIRK2/3 in GnRH neurons. *In vivo*, double-labeled OFQ/POMC fibers were found in the vicinity of GnRH neurons, and OFQ fibers apposed GnRH neurons. Together, this study brings to light a potent neuromodulator of GnRH neurons.

## Significance Statement

Fertility is controlled centrally by neurons secreting gonadotropin-releasing hormone (GnRH) and critically relies on their pulsatile secretory profile. GnRH pulses depend on kisspeptin neurons located in the arcuate nucleus. However, kisspeptin provides a long-lasting stimulation, and how GnRH neurons return to baseline electrical activity is unknown. Here, we show nociceptin/orphanin-FQ potently inhibits GnRH neurons. The signaling pathway involves the receptor, opioid receptor like 1, and downstream effectors G_i/o_-proteins and G_i/o_-protein-coupled inwardly rectifying potassium (GIRK) channels. Notably, the GIRK channels in GnRH neurons exhibit a specific subunit composition, GIRK2/3. Together, these data identify a new messenger in modulating reproductive function.

## Introduction

Fertility relies on the capacity of gonadotropin-releasing hormone (GnRH) neurons to convert a wide range of cues from the CNS into a final hormonal signal to the pituitary, which subsequently controls the gonads. Steroids from the gonads feedback to the CNS providing the physiologic signals for coordinated communication along the GnRH–pituitary–gonadal axis and thus, reproductive success. Notably, GnRH is secreted in a pulsatile fashion (i.e., it must be initiated and stopped). Kisspeptin neurons are key players in the model for pulsatile release of GnRH (Mittelman-Smith et al., 2012; Navarro, 2012; Goodman et al., 2013). In this model, the action of stimulatory neurokinin B and inhibitory dynorphin A drives kisspeptin neurons into an oscillating firing mode that leads to pulsatile kisspeptin release. The phasic activity of GnRH neurons is subsequently triggered by kisspeptin. While dynorphin A ends the kisspeptin release and therefore removes the excitation of GnRH neurons, exogenous kisspeptin triggers a long-lasting excitation in GnRH neurons, both at the cell body and at the terminal (Han et al., 2005; Constantin et al., 2009, 2013; Iremonger et al., 2017). The endogenous kisspeptin released after stimulation of the anteroventral periventricular nucleus (AVPV) also evokes the classical long-lasting effect (Liu et al., 2011). GnRH secretion seems to end while the kisspeptin induced excitation still persists (Glanowska and Moenter, 2015). To date, the identity of the molecule that terminates the bout of electrical activity and allows GnRH neurons to return to baseline electrical activity is unknown.

High levels of the nociceptin/orphanin FQ (OFQ) receptor ORL1 (opioid receptor like 1) are found in the preoptic area (POA) and the anterior hypothalamic area where GnRH neurons reside (Houtani et al., 2000). *In vitro*, OFQ directly hyperpolarizes GnRH neurons in the arcuate nucleus (ARC; guinea pigs; Wagner et al., 1998), inhibits forskolin-evoked GnRH release from hypothalamic fragments (male rats; Dhandapani and Brann, 2002) and the spontaneous release of GnRH from mediobasal hypothalamus fragments [ovariectomized (OVX) rats; An et al., 2005]. *In vivo*, OFQ push–pull infusion inhibits GnRH release from the POA (OVX rats; An et al., 2005) and intracerebroventricular OFQ inhibits GnRH release from the median eminence (OVX rats; An et al., 2005, 2009). OFQ intracerebroventricular injections also decrease circulating luteinizing hormone (LH) levels (OVX rats; An et al., 2005) and blunts the preovulatory surge (OVX+estradiol/progesterone rats; An et al., 2007). Pharmacology or RT-PCR analysis support the effects being ORL1 dependent. Together, these data suggest that ORL1 is expressed on GnRH neurons, and OFQ may act as an inhibitory neuromodulator (Mollereau and Mouledous, 2000).

The ARC contains a variety of neuropeptides (Chronwall, 1985) including OFQ (Maolood and Meister, 2010; Campbell et al., 2017). Proopiomelanocortin (POMC) neurons are one of the ARC neuronal subpopulations that coexpress OFQ [male rats (Maolood and Meister, 2010); ewes (Nestor et al., 2013]. POMC neurons express both ERα (Jirikowski et al., 1986; Xu et al., 2011) and progesterone receptor (Fox et al., 1990). ARC-specific (Yeo and Herbison, 2014) and POMC-specific (Xu et al., 2011) depletion of ERα disrupted LH responses to estrogen-negative feedback in female mice. Afferents from the ARC (Mezey et al., 1985; Wintermantel et al., 2006), and specifically from POMC neurons (Leranth et al., 1988; Simonian et al., 1999), are present in the vicinity of GnRH cell bodies in the preoptic area, and the response of GnRH neurons to the POMC-derived peptides, α-melanocyte-stimulating hormone and β-endorphin, clearly supports a functional transsynaptic link between POMC neurons and GnRH neurons (Roa and Herbison, 2012). In contrast, the literature indicates that the POMC neurons in the nucleus of the solitary tract are unlikely to play a role in the control of fertility (Roa, 2013). In OVX ewes, the infusion of an ORL1 antagonist in the ARC does not affect LH pulses (Goodman et al., 2013) but, intracerebroventricular ORL1 antagonists increase LH levels under estradiol/progesterone supplementation, suggesting that OFQ may participate in progesterone-negative feedback (Nestor et al., 2013).

The present study shows *Orl1* transcripts in GnRH cells and that OFQ inhibits GnRH neuronal activity, without GABAergic and glutamatergic inputs, and suppresses kisspeptin-10-evoked excitation. The signaling pathway, initiated by ORL1, was identified as G_i/o_-type G-protein coupling mediated by G-protein-coupled inwardly rectifying potassium (GIRK) channels, most likely GIRK2/3 heteromers. In addition, we found POMC fibers coexpressing OFQ in the vicinity of GnRH neurons and OFQ fibers contacting GnRH neurons in the POA, suggesting that these fibers might originate from POMC neurons in the ARC. Together, these data highlight N/OFQ as a potent *in vivo* inhibitory signal to GnRH neurons in the mouse.

## Materials and Methods

### Animals

All procedures were approved by National Institute of Neurologic Disorder and Stroke, Animal Care and Use Committee, and were performed in accordance with National Institutes of Health (NIH) guidelines. Mice were maintained under 12 h light/dark lighting conditions, with food and water available *ad libitum*. Embryos were collected from timed-pregnant NIH Swiss mice at embryonic day 11.5, and nasal explants were generated (see below) for PCR and calcium imaging experiments. For *in vivo* immunocytochemistry, adult intact GnRH-green fluorescent protein (GFP; Mouse Genome Informatics ID 6158458) male mice (Spergel et al., 1999) were used for GnRH/OFQ and GnRH/POMC staining, and adult intact C57BL/6 male mice were used for POMC/OFQ staining. Adult intact GnRH-GFP male mice were also used to generate brain slices for electrophysiological experiments.

### GnRH cells maintained in nasal explants

Explants were cultured as previously described (Fueshko and Wray, 1994). Briefly, gestational day 11.5 embryos (undetermined sex) were obtained from time-mated pregnant NIH Swiss mice. Nasal pits were dissected under aseptic conditions in Gey’s Balanced Salt Solution (Life Technologies) supplemented with glucose (Sigma-Aldrich). Explants were adhered onto coverslips by a plasma (Cocalico Biologicals)/thrombin (Sigma-Aldrich) clot and maintained in a defined serum-free medium (SFM) in a humidified atmosphere at 37°C with 5% CO_2_. On culture day 3, SFM was replaced by fresh SFM containing fluorodeoxyuridine (2.3 µm; Sigma-Aldrich) for 3 d to inhibit the proliferation of dividing olfactory neurons and non-neuronal explant tissue. On culture day 6, and every 2 d afterward, the medium was changed with fresh SFM.

### PCR on cDNA from single GNRH neurons maintained in explants

Poly(A)-amplified cDNA libraries were generated from single GnRH neurons using two different techniques (Kramer, 2002; Bosch et al., 2013). Every single-cell cDNA pool generated was first tested by PCR for GnRH (Giacobini et al., 2004). Cellular material without reverse transcriptase, and no cellular material (water), served as negative controls. Specific primers were designed in the 3´-untranslated region of the genes encoding ORL1 within 300 bp before the polyadenylation site. All designed primers were screened using NCBI BLAST (Basic Local Alignment Search Tool; Johnson et al., 2008) to ensure specificity. For each reaction, 1× PCR buffer, 2 mm MgCl2, 250 µm each deoxynucleotide mix (Life Technologies), 125–250 nm forward primer, 125–250 nm reverse primer, and 2.5 U AmpliTaq Gold (Life Technologies) were added to 1–3 µl template cDNA. PCR was performed as follows: initial 10 min denaturation (94°C); 40–50 cycles with denaturation 30 s (94°C); annealing for 30 s (55–66°C) and extension for 2 min (72°C); followed by 10 min postelongation at 72°C. Amplified products were run on a 1.5% agarose gel. Specific bands of the predicted size were observed in control total brain, whereas no bands were seen in water. The sequences of the primers are listed in Table 1.

**Table 1:** Primer sequences

Gene (NCBI/GenBank ID) Reference Sequence)	Primers sequences (5´ to 3´)	Annealing temperature	Product size
	
GnRH-1 (NM_008145.2)	CTG ATG GCC GGC ATT CTA CTG C	66°C	220 bp
	CCA GAG CTC CTC GCA GAT CCC		
Opioid receptor-like 1 (NM_011012.5)	CAT GCC ATG CAG AAC CCA G	55°C	209 bp
	AGG GCT AGC TAC ATG CAC GA		

### Calcium imaging

**Figure 1:**
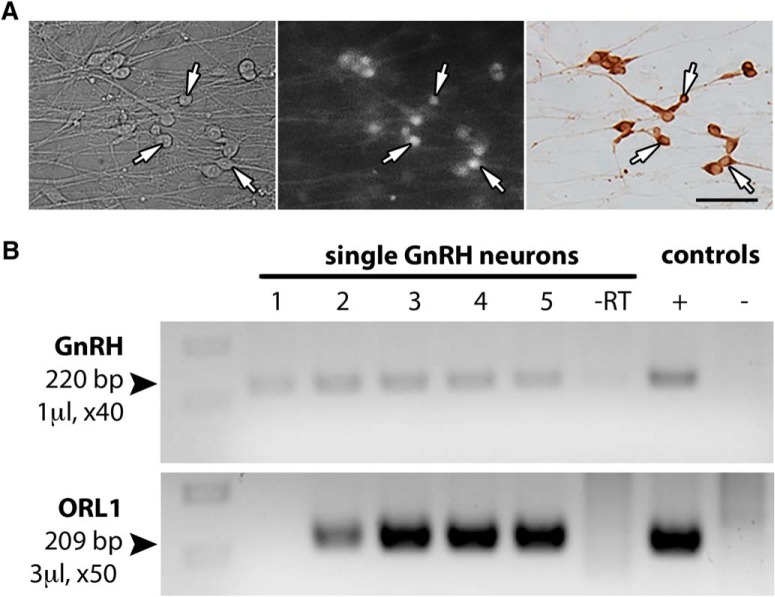
GnRH neurons express the N/OFQ receptor ORL1. ***A***, High**-**magnification image of GnRH neurons (arrows) recorded with calcium imaging. Initially identified by their bipolar morphology (left), GnRH neurons loaded with the calcium-sensitive dye Calcium Green**-**1 AM (middle) were imaged. The phenotype of cells was confirmed *post hoc* by immunocytochemistry (right). Scale bar, 50 µm. ***B***, Embryonic GnRH neurons express *Orl1*. Transcripts for the receptor ORL1 were found in single GnRH neurons. Adult brain was used as positive control and water and reverse transcriptase were used as negative controls, respectively. The volume of template and number of cycles are as indicated.

GnRH neurons exhibit calcium oscillations that correlate with bursts of action potentials (APs; Constantin and Wray, 2008); therefore, calcium imaging was used as a reflection of GnRH neuronal activity. Experiments were performed as previously described (Constantin et al., 2009). Explants were used between 6 and 11 d in culture (Fig. 1*A*). Briefly, Calcium Green-1 AM (Life Technologies) was dissolved at 2.7 mm in dimethylsulfoxide containing 20% pluronic F-127 (Life Technologies), then diluted down to 13.5 µm in SFM (loading solution), aliquoted, and kept frozen until use. Explants were incubated in warm loading solution for 20 min at 37°C in a 5% CO_2_ humidified incubator. After washes in fresh SFM, explants were mounted in a perfusion chamber (Warner Instruments) and continuously perfused at a rate of ∼300 μl/min. Calcium imaging experiments were performed at 25°C. Calcium Green-1 was visualized using an inverted microscope (Eclipse TE2000-E, Nikon), through a 20× fluorescence objective [Fluor 20×; numerical aperture (NA), 0.75; working distance (WD), 1.0 mm] and a charge-coupled device camera (QImaging) connected to a computer. Time-lapse recording was piloted by iVision imaging software (Scanalytics), and pictures were acquired every 2 s. Excitation wavelengths were provided with a medium-width excitation bandpass filter at 465–495 nm, and emission was monitored through a 40 nm bandpass centered on 535 nm. Calcium imaging recordings were divided into periods. The treatment period was preceded by a control period in SFM, followed by a washout period. When possible, drugs were sequentially added through multiple treatment periods. When a drug required a long pretreatment [i.e., pertussis toxin (PTX) or tertiapin-Q (TPNQ)], recordings were started during the last 10 min of pretreatment to determine the basal level of GnRH neuronal activity. All recordings were terminated with a 40 mm KCl stimulation to ensure the viability of the cells. The changes in fluorescence over time were measured in single, phase bright, bipolar cells with iVision and analyzed with MATLAB (MathWorks) as previously described (Constantin and Wray, 2008). The phenotype of these bipolar cells, defined as regions of interest, was confirmed using chromogen immunocytochemistry against GnRH. Due to the heterogeneity of GnRH neuronal population, a large number of cells were sampled for each paradigm. The individual cells (*n*) originating from at least three explants (*N*) independently recorded were combined for each paradigm. Explants (*N* = 65) contained on average 29.5 ± 2.0 identified GnRH neurons in a recording field.

### Immunocytochemistry for GnRH

After calcium imaging, explants (6–11 d) were fixed for 30 min with 0.1 m PBS, pH 7.4, containing 4% formaldehyde at room temperature. After a few washes in PBS, explants were incubated in a blocking solution (10% normal horse serum plus 0.3% Triton X-100) for 1 h, and washed several times in PBS. The explants were incubated at 4°C overnight in the primary antibody (rbGnRH; Wray et al., 1989b; Table 4). The next day, explants were washed in PBS, incubated for 1 h with biotinylated secondary donkey anti-rabbit antibody (1:500 in PBS/0.3% Triton X-100; Vector Laboratories), washed in PBS, and processed for avidin–biotin horseradish peroxidase/3,3´-diaminobenzidine (Fig. 1*A*).

### Immunofluorescent labeling

Three explants (6–11 d) were fixed, washed, and blocked as described above. The explants were then incubated (4°C overnight) in the GIRK2 primary antibody, which has been shown to give no signal in GIRK2 knock-out mice (Marron Fernandez de Velasco et al., 2017). The next day, explants were washed in PBS, and the first primary antibody was visualized using Alexa Fluor 488-conjugated secondary donkey anti-rabbit antibody (1 h, 1:1000 in PBS/0.3% Triton X-100). Explants were then washed, fixed for 10 min, washed, and incubated in the second primary antibody [mouse GnRH (mGnRH); 2 d]. After washing (PBS), the second primary antibody was visualized using Alexa Fluor 555-conjugated secondary donkey anti-mouse antibody (1:1000 in PBS/0.3% Triton X-100). After several washes in PBS and water, explants were coverslipped with an anti-fade mounting solution. Controls (*N* = 3) in which the anti-GIRK2 primary antibody was omitted resulted in only background staining.

Adult mice, anesthetized with isoflurane then killed with an intraperitoneal overdose of ketamine (20 mg/20 *g*), were transcardially perfused with 0.1 m PBS then 4% formaldehyde in PBS. The brains were removed and postfixed in the same fixative (1 h), then transferred to a 30% sucrose-PBS solution overnight. The next day, the brains were frozen in dry ice and kept at −80°C until sectioning. Four sets of coronal sections (40 µm) were cut with a sliding microtome and kept at −20°C in cryoprotectant (Hoffman et al., 2008) until staining. After washes in PBS, sections were incubated for 1.5 h in a blocking solution (see above), washed several times in PBS, and incubated in primary antibodies. Three different adult males were used for each staining.

### OFQ or POMC staining and GFP staining in GnRH-GFP mice

Sections were incubated at 4°C (2 nights) in the first primary antibody (OFQ or POMC). The next day, sections were washed in PBS, incubated for 1.5 h with Alexa Fluor 555-conjugated secondary donkey anti-rabbit antibody (1:1000 in PBS/0.3% Triton X-100). After several washes in PBS, sections were rapidly fixed with PBS containing 4% formaldehyde, washed, and incubated at 4°C (for 2 nights) in the second primary antibody (GFP). The next day, sections were washed in PBS and incubated for 1.5 h with Alexa Fluor 488-secondary donkey anti-chicken antibody (1:1000 in PBS/0.3% Triton X-100). After several washes in PBS and water, sections were coverslipped with an anti-fade mounting solution.

### OFQ and POMC staining

To avoid cross-reactivity between two primary antibodies raised in a rabbit, staining was performed as described previously (Shindler and Roth, 1996; Hoffman et al., 2008). Sections were incubated at 4°C (for 2 nights) in the first primary antibody (POMC; Elkabes et al., 1989). The next day, sections were washed in PBS, incubated with biotinylated secondary donkey anti-rabbit antibody (1 h, 1:1000 in PBS/0.3% Triton X-100). After washes in PBS, sections were incubated in avidin–biotin complex (1 h, 1:500 in PBS/0.3% Triton X-100), washed in PBS, and incubated in tyramide (20 min, 1:200 in PBS/0.3% Triton X-100/0.005% H_2_O_2_; PerkinElmer). Then sections were washed, incubated with streptavidin Texas Red (1.5 h,1:200 in PBS/0.3% Triton X-100; PerkinElmer), washed, fixed (10 min), washed, and incubated (2 d) in the second primary antibody (OFQ). Sections were then washed in PBS, incubated with Alexa Fluor 488-conjugated donkey anti-rabbit antibody (1.5 h, 1:1000 in PBS/0.3% Triton X-100), washed in PBS, and mounted and coverslipped with an anti-fade mounting solution. For triple labeling (one series/animal), an antigen retrieval method was used before initiating the immunocytochemistry (Jiao et al., 1999), and staining for GnRH was performed after staining for POMC and OFQ using an GnRH antibody raised in chicken. Sections were incubated for 2 d, washed, and visualized with Alexa Fluor 647-conjugated donkey anti-chicken antibody.

All immunofluorescent pictures were taken using spinning disk confocal (Yokogawa) microscopy (Eclipse TE-200, Nikon) though a 60× water-immersion objective (Nikon Plan Apo 60×; NA, 1.2; WD, 0.27 mm), captured with a high-sensitivity camera (EM-CCD, Hamamatsu Photonics) and presented as a flattened confocal stack or a single focal plan.

The specificity of the OFQ antibody was confirmed by the presence of a signal on previous documented structures, as follows: (1) suprachiasmatic nucleus, (2) trigeminal ganglion, and (3) dorsal root ganglion (Fig. 8-1).

### Electrophysiology

Male mice were chosen to avoid the possible influence of fluctuating circulating steroids. GnRH-GFP mice were killed at ∼1030 h by cervical dislocation then decapitated. The brain was removed from the skull, glued to the vibratome plate, submerged with iced-cold low [Ca]/high [Mg] (0.5 and 6 mm, respectively) artificial CSF (aCSF), and bubbled with 95% O_2_/5% CO_2_. Conventional coronal sections (200 µm) were cut using a vibratome (VT1000S, Leica). After sectioning, slices were incubated at 30°C in normal aCSF containing, as follows: 118 mm NaCl, 3 mm KCl, 2.5 mm CaCl2, 1.2 mm MgCl2, 10 mm HEPES, 25 mm NaHCO3, and 11 mm d-glucose, pH 7.3, bubbled with 95% O_2_/5% CO_2_. Individual slices were transferred into a recording chamber mounted on an upright microscope (Eclipse FN1, Nikon) and continuously superfused with oxygenated normal aCSF maintained at 28–30°C at a rate of ∼2 ml/min (Constantin et al., 2012). Individual GnRH neurons were identified with fluorescence (20 nm narrow bandpass EGFP filter centered at 480 nm) using a 40× water-immersion objective (40×/0.80 W; WD, 2.0 mm; Nikon). Visualized with a charge-coupled device camera (Retiga EXi Blue, QImaging) and piloted by the open source software Micro-Manager version 1.4, the neurons were patched under fluorescence and differential interference contrast. The pipettes (3–5 MΩ) were backfilled with aCSF. Electrophysiological recordings were acquired with a Multiclamp 700B amplifier (Molecular Devices) using a low-pass filter at 10 kHz and digitized by a Digidata (1550) analog-to-digital converter at 10 kHz (Molecular Devices).

### Drugs

Cesium (Ca), barium [Ba; both broad-spectrum blockers of inwardly rectifying potassium (K_ir_) channels], naringin (GIRK channel activator), phorbol 12-myristate 13-acetate [PMA; protein kinase C (PKC) activator], and baclofen (BAC; GABA_B_ receptor agonist) were purchased from Sigma-Aldrich. 3-isobutyl-1-methylxanthine (IBMX; inhibitor of phosphodiesterase), forskolin (FSK; activator of adenylyl cyclase), 6-Cyano-7-nitroquinoxaline-2,3-dione (CNQX; AMPA/kainate receptor antagonist), d(−)-2-amino-5-phosphonopentanoic acid (d-AP5; NMDA receptor antagonist), bicuculline (BIC; GABA_A_ receptor antagonist), TPNQ (blocker of GIRK channels), UFP-101 (UFP) and SB 162111 (SB; two selective antagonists for ORL1 receptor), PTX (uncoupling G_i/o_-protein-coupled receptor), ML297 (GIRK channel activator) and human kisspeptin-10 [kp-10; Tyr-Asn-Trp-Asn-Ser-Phe-Gly-Leu-Arg-Phe-NH2] were purchased from Tocris Bioscience. Nociceptin/orphanin FQ (1-13)-NH2 [OFQ; Phe-Gly-Gly-Phe-Thr-Gly-Ala-Arg-Lys-Ser-Ala-Arg-Lys-NH2] was purchased from Phoenix. All stock solutions (1000× or 500×) were stored at −20°C and diluted prior to each experiment at the specified concentration in SFM.

Note that throughout the calcium-imaging and electrophysiology result sections, OFQ refers to the truncated form, OFQ(1-13)-NH2, exogenously applied.

### Statistical analysis

For calcium imaging, comparisons of the frequencies of calcium oscillations (in peaks/min) were performed using a repeated measurement one-way ANOVA with Greenhouse–Geisser correction, and *post hoc* Sidak’s multiple-comparisons test between two consecutive recording periods for paradigms (Table 2). A paired Student’s *t* test was used for two-period paradigms (Table 2). The strength of inhibition was determined for each cell and was expressed as the percentage of the pre-OFQ period as follows: [(peaks/min before OFQ) − (peaks/min during OFQ)]/(peaks/min before OFQ) × 100. Comparisons of the strength of inhibition between groups of cells were performed with one-way ANOVA, and *post hoc* Dunnett’s multiple-comparisons test, using OFQ as the reference. In the Results section and figures, the frequencies of calcium oscillations are expressed as the mean ± SEM, and *n* and *N* represent the number of cells and explants recorded, respectively.

**Table 2:** Frequencies of calcium oscillations in GnRH neurons

		Frequencies (peaks/min)				Cells (*n*)	Explants (*N*)
	Paradigms with OFQ(1-13)	Period 1	Period 2	Period 3	Period 4		
							
a	SFM – OFQ [10 nm] – SFM	1.49 ± 0.07	0.27 ± 0.03* p < 0.0001 *d* = 2.1557	0.40 ± 0.06 *p* = 0.0253 *d* = 0.2511		102	3
b	SFM – OFQ [1 nm] – SFM	1.69 ± 0.12	0.39 ± 0.04* p < 0.0001 *d* = 1.9704	0.87 ± 0.08* p < 0.0001		69	3
c	SFM – OFQ [100 pm] – SFM (Fig. 2*A*)	1.44 ± 0.07	0.32 ± 0.03* p < 0.0001	0.75 ± 0.07* p < 0.0001	-	144	5
d	SFM – OFQ [10 pm] – SFM	1.53 ± 0.09	0.59 ± 0.08* p < 0.0001	1.15 ± 0.10* p < 0.0001	-	78	3
e	SFM – AAB – AAB + OFQ – SFM (Fig. 2*B*)	2.14 ± 0.09	1.36 ± 0.09* p < 0.0001	0.48 ± 0.06* p < 0.0001	1.65 ± 0.09* p < 0.0001	113	3
f	SFM – UFP – UFP + OFQ – SFM (Fig. 3*A*)	1.47 ± 0.08	1.42 ± 0.07 *p* = 0.4235	1.19 ± 0.07* p < 0.0001	1.14 ± 0.07 *p* = 0.6823	113	3
g	SFM – SB – SB + OFQ – SFM (Fig. 3*B*)	1.86 ± 0.06	1.95 ± 0.05 *p* = 0.0532	1.42 ± 0.06* p < 0.0001	1.83 ± 0.06* p < 0.0001	244	6
h	PTX pretreated: SFM – OFQ – SFM (Fig. 4*A*)	2.26 ± 0.11	1.91 ± 0.12* p < 0.0001	2.23 ± 0.12* p < 0.0001		89	3
i	SFM – IBMX + FSK – IBMX + FSK + OFQ – SFM (Fig. 4*B*)	1.68 ± 0.07	1.62 ± 0.07 *p* = 0.8887	0.46 ± 0.04* p < 0.0001	1.10 ± 0.07* p < 0.0001	161	3
j	10 min Cs pretreated: Cs – Cs + OFQ (Fig. 4*C*)	1.74 ± 0.06	1.15 ± 0.06* p < 0.0001			169	5
k	20 min Ba pretreated: Ba – Ba + OFQ (Fig. 4*D*)	1.71 ± 0.11	1.16 ± 0.11* p = 0.0035			36	4
l	SFM – TPNQ – TPNQ + OFQ – SFM (Fig. 5*A*)	2.22 ± 0.11	2.32 ± 0.10 *p* = 0.416	0.64 ± 0.07* p < 0.0001	1.81 ± 0.13* p < 0.0001	80	3
m	SFM – naringin	2.04 ± 0.15	2.00 ± 0.14 *p* = 0.6761			50	4
n	SFM – ML297	1.85 ± 0.08	1.83 ± 0.08 *p* = 0.6346			118	3
o	SFM – PMA – PMA + OFQ – SFM (Fig. 5*C*)	1.40 ± 0.10	1.62 ± 0.08 *p* = 0.0122	1.37 ± 0.08 *p* = 0.0914	1.47 ± 0.09 *p* = 0.5211	118	4
p	SFM – BAC – SFM	2.04 ± 0.10	0.30 ± 0.04* p < 0.0001	1.64 ± 0.10* p < 0.0001		89	4
q	SFM – TPNQ – TPNQ + BAC – SFM (Fig. 5*B*)	1.80 ± 0.14	2.02 ± 0.13 *p* = 0.0068	0.13 ± 0.04* p < 0.0001	1.71 ± 0.99* p < 0.0001	53	3
r	SFM – PMA – PMA + BAC – SFM (Fig. 5*D*)	2.10 ± 0.12	2.19 ± 0.10 *p* = 0.8219	1.81 ± 0.12* p = 0.0167	2.08 ± 0.12 *p* = 0.1482	92	3

Concentrations used: OFQ, 100 pm (unless stated); AABs (BIC, 20 µm; CNQX, 10 µm; d-AP5, 10 µm); UFP, 10 nm; SB, 10 nm; PTX, 250 ng/ml; IBMX, 10 µm; FSK, 1 µm; Cs, 5 mm; Ba, 400 µm; TPNQ, 500 nm; naringin, 500 µm; ML297, 10 µm; BAC, 10 µm; PMA, 50 nm. Data are expressed as the mean ± SEM.

* Significant difference compared with the previous period (period 2 with period 1 or period 3 with period 2 or period 4 with period 3) using repeated-measures one-way ANOVA, followed by Sidak’s multiple-comparisons test (*p* < 0.01). For rows j, k, m, and n, a paired Student’s *t* test was used to compare period 2 with period 1 (*p* < 0.01).

For electrophysiology, the OFQ-induced inhibition was determined as follows: APs were detected with Clampfit 10 on continuous recordings, and the firing frequency (in Hz) was determined by summing APs into 1 s bins. The average firing frequency was calculated over the last minute of each recording period, control before OFQ, during OFQ and washout after OFQ (<6 min). When firing was not regained spontaneously, kisspeptin-10 was applied to validate the period of silence. Periodograms (in Hz) were performed by summing APs into 10 s bins. A Student’s paired *t* test was performed between two consecutive recording periods. *n* and *N* represent the number of cells and the number of animals the cells are recorded from, respectively. In calcium imaging, significant differences were defined by a *p* value <0.01 due to the large number of cells that can be sampled; in electrophysiology, significant differences were defined by a *p* value <0.05.

## Results

RT-PCR analysis revealed the presence of transcripts coding for the ORL1 gene (*n* = 14 and 19) in single GnRH neurons maintained in nasal explants using two different single-cell RT-PCRs (*n* = 15 and 19; Fig. 1*B*).

### Orphanin FQ (1-13) directly inhibits GnRH neurons maintained in explants

**Figure 2.**
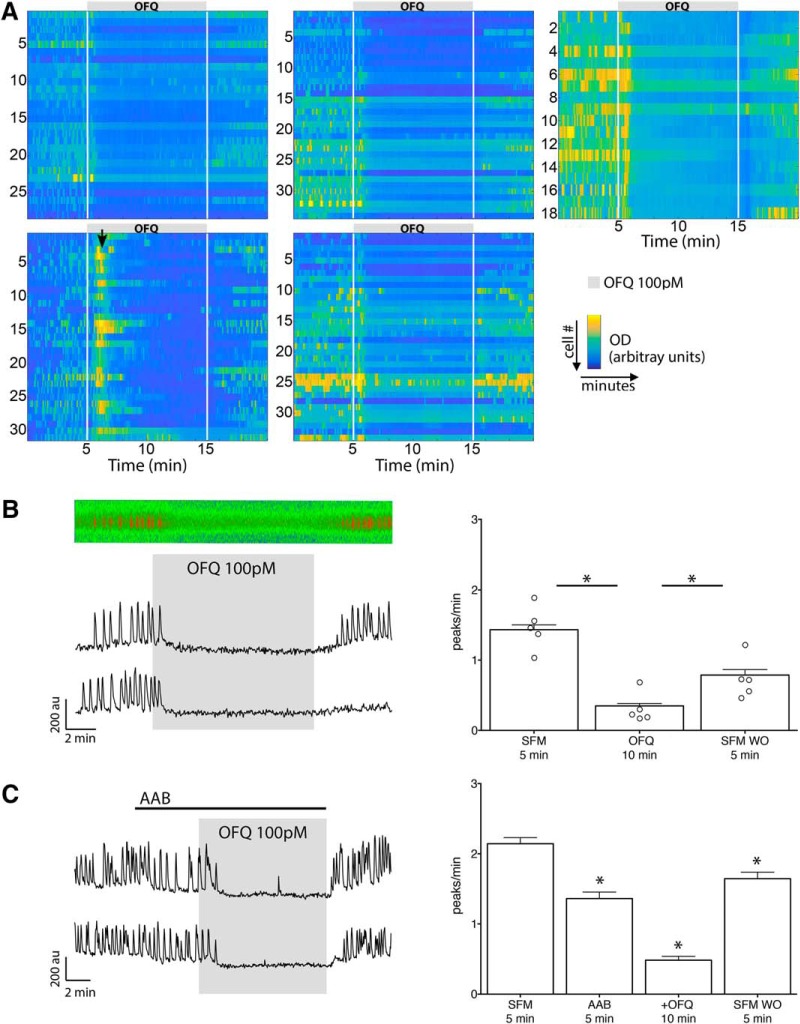
OFQ inhibits GnRH neuron calcium oscillation, independent of GABAergic and glutamatergic inputs. ***A***, Heatmaps of changes in levels of intracellular calcium in cells recorded simultaneously during the paradigm in ***B***, independently repeated in five different explants. Each row represents changes in a single cell. White lines indicate the time of drug application. Note the consistency of the response to OFQ within each explant and across explants (Fig. 2-1). ***B***, Left, Calcium-imaging recordings of explants showing OFQ (100 pm) evoked a potent decrease in the frequency of calcium oscillations in GnRH neurons (kymograph from the cell shown on the top trace). Right, Empty circles, Summary data showing the average frequency of calcium oscillations for each explant. To minimize the impact of the cell-to-cell heterogeneity, the values from all individual cells originating from the five explants in ***A*** were combined. Bars, Summary data showing the average frequency of calcium oscillations in all GnRH neurons tested during the control (SFM), treatment (OFQ), and the washout (SFM WO) periods (*n* = 144, *N* = 5). au, Arbitrary unit. *Statistical significance between two consecutive periods. ***C***, Left, The OFQ-evoked inhibition persisted in the presence of AABs (BIC, 20 µm; CNQX, 10 µm; d-AP5, 20 µm). Right, Summary data showing the average frequency of calcium oscillations in all GnRH neurons tested during the control (SFM), pretreatment (AAB), treatment (+OFQ), and washout (SFM WO) periods (*n* = 113, *N* = 3).

OFQ was applied to 6-11 d explants at four different concentrations (10 nm, 1 nm, 100 pm, and 10 pm). All doses inhibited GnRH neuronal activity (Table 2, rows a–d, Fig. 2). The mean response of individual explants was quantified to assess whether the OFQ response might be sex specific. OFQ evoked a potent inhibition of GnRH neurons in every explant tested (*N* = 20, from all four doses combined; Fig. 2-1); therefore, the sex of the embryo from which the explant was generated is highly unlikely to define the responsiveness to OFQ. At 100 pm, ∼90% of the cells showed a ≥50% inhibition with OFQ, and the inhibition was not correlated with cell location within the explant periphery (Fig. 2*A*). The dose of 100 pm was chosen for all subsequent experiments, as it was the smallest dose with robust effects (Fig. 2*B*).

10.1523/ENEURO.0161-18.2018.f2-1 Figure 2-1 The response to OFQ is consistent throughout the explants. The graph represents the average inhibition calculated per individual explant (*N* = 14) at the four OFQ doses tested. All explants (unsexed) displayed a similar level of inhibition, ruling out the influence of gender in the OFQ inhibition. Download Figure 2-1, TIF file.

In explants, the spontaneously occurring GnRH neuronal activity is mainly regulated by GABAergic and glutamatergic inputs (Constantin et al., 2010). GABA_B_ receptors are not endogenously activated in this model at 6–11 d *in vitro* (Constantin et al., 2010), and metabotropic glutamatergic receptors are unlikely to play a major role on GnRH neurons (Chu and Moenter, 2005; Dumalska et al., 2008). Thus, the application of OFQ was repeated in the presence of an amino acid blocker (AAB) cocktail (BIC, 20 µm; CNQX, 10 µm; d-AP5, 10 µm) to inhibit GABA_A_ receptors and ionotropic glutamatergic inputs. The OFQ inhibition persisted in the presence of AAB (Table 2, row e), demonstrating a direct effect of OFQ on GnRH neurons (Fig. 2*C*). Approximately 75% of the cells showed a ≥50% inhibition with AAB plus OFQ.

### The receptor of orphanin FQ, ORL1, mediates the OFQ inhibition

**Figure 3.**
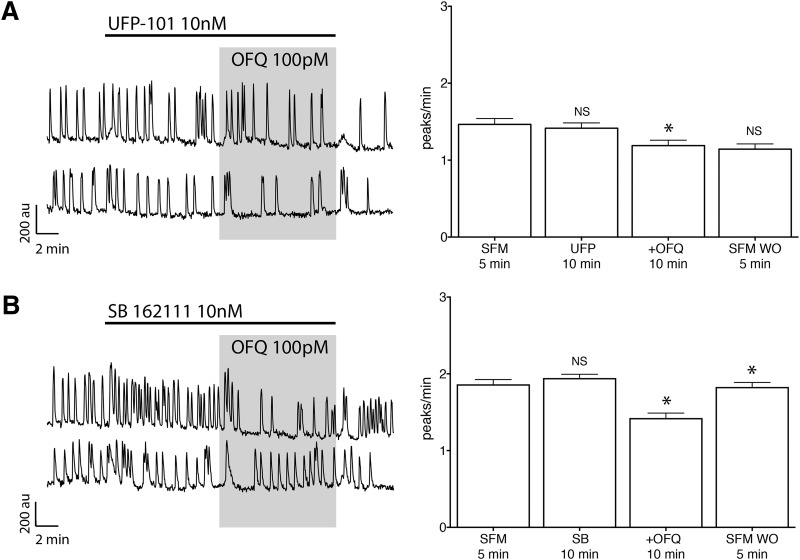
OFQ-evoked inhibition of GnRH neuronal activity is mediated by the receptor ORL1. ***A***, Left, Calcium imaging recordings showing OFQ-evoked largely reduced inhibition in the presence of the ORL1 antagonist UFP-101. Right, Summary data showing the average frequency of calcium oscillations in all GnRH neurons tested during the control (SFM), pretreatment (UFP-101), treatment (+OFQ), and the washout (SFM WO) periods (*n* = 113, *N* = 3). au, Arbitrary unit. *Statistical significance between two consecutive periods. ***B***, Left, The ORL1 antagonist SB also reduced the OFQ-evoked inhibition. Right, Summary data showing the average frequency of calcium oscillations in all GnRH neurons tested during the control (SFM), pretreatment (SB 162111), treatment (+OFQ), and washout (SFM WO) periods (*n* = 244, *N* = 6).

UFP is a selective antagonist for ORL1 receptors (Calo et al., 2002). Pretreatment of GnRH neurons with UFP (10 nm) did not completely prevent OFQ-induced inhibition (Table 2, row f, Fig. 3*A*) but dramatically attenuated it (Table 3, rows a and b), with only ∼12% of the cells showing a ≥50% inhibition. The effectiveness of UFP-101 is shown with cells losing their ability to respond to the second dose of OFQ (see Fig. 6*A*,*B*). The experiment was repeated with SB, another selective antagonist for ORL1 receptors. Pretreatment of GnRH neurons with SB (10 nm) also attenuated OFQ inhibition (Tables 2, row g, 3, rows a and c, Fig. 3*B*) with ∼35% of the cells showing a ≥50% inhibition. These data support the binding of OFQ to ORL1 receptors in GnRH neurons. Note that UFP and SB did not have any effect on their own (Table 2, rows f and g).

**Table 3:** Inhibition of GnRH neurons

	Paradigms with OFQ(1-13)	Period (with OFQ) − Period (without OFQ)/Period (with OFQ) (change in %)	Cells (*n*)	Explants (*N*)
	
a	SFM – OFQ	−75.6 ± 2.0	144	5
b	UFP – UFP + OFQ	−17.3 ± 3.3* (*p* < 0.0001)	112	3
c	SB – SB + OFQ	−29.5 ± 2.5* (*p* < 0.0001)	244	6
d	PTX pretreated: SFM – OFQ	−17.4 ± 4.4* (*p* < 0.0001)	88	3
e	IBMX + FSK – IBMX + FSK + OFQ	−68.2 ± 2.6 (*p* > 0.9864)	161	3
f	Cs – Cs + OFQ	−28.6 ± 3.6* (*p* < 0.0001)	168	5
g	Ba – Ba + OFQ	−12.0 ± 4.9* (*p* < 0.0001)	36	4
h	TPNQ – TPNQ + OFQ	−72.9 ± 2.5 (*p* > 0.9999)	80	3
i	PMA – PMA + OFQ	18.7 ± 16.8* (*p* < 0.0001)	113	4
j	SFM – BAC	−86.2 ± 1.9	88	4
k	TPNQ – TPNQ + BAC	−93.5 ± 1.5 (*p* = 0.9206)	53	3
l	PMA – PMA + BAC	19.9 ± 20.5* (*p* < 0.0001)	92	3

Concentrations used: OFQ, 100 pm; UFP, 10 nm; SB, 10 nm; PTX, 250 ng/ml; IBMX, 10 µm; FSK, 1 µm; Cs, 5 mm; Ba, 400 µm; TPNQ, 500 nm; PMA, 50 nm; BAC, 10 µm. Data are expressed as the mean ± SEM.

* Significant difference (*p* < 0.01), using one-way ANOVA, followed by Dunnett’s multiple-comparisons test using OFQ (row a) as a reference for rows b–i or BAC (row j) as a reference for rows k and l).

**Table 4:** Antibodies

Peptide/protein Target	Name of antibody (RRID when available)	Manufacturer, catalog number, and/or name of individual providing the antibody	Species raised in; monoclonal or polyclonal	Dilution used	Tissue; labeling
			
rbGnRH	SW-1 (AB_2629221)	S. Wray	Rabbit; polyclonal	1:3000	Explants; DAB (Fig. 1*A*)
GIRK2	APC-006 (AB_2040115)	Alomone Labs	Rabbit; polyclonal	1:800	Explants; DAR-488 (Fig. 7)
mGnRH	F1D3C5 (n/a) + SMI-41 (AB_10123893)	A. Karande + Abcam	Mouse; monoclonal	1:4000 +1:6000	Explants; DAM-555 (Fig. 7)
N/OFQ	RA10106 (AB_2737116)	Neuromics	Rabbit; polyclonal	1:3000	Sections; DAR-555 (Fig. 8) Sections; DAR-488 (Fig. 8-1*A*,*B*; Figs. 9, 10*D–F*) Sections; DAB (Fig. 8-1*C*,*D*)
GFP	ab13970 (AB_300798)	Abcam	Chicken; polyclonal	1:2000	Sections; DAC-488 (Figs. 8, 10*A–C*)
POMC	DP4 (n/a)	P. Loh	Rabbit; polyclonal	1:2500	Sections; avidin-Texas Red (Figs. 9, 10*D–F*) Sections; DAR-555 (Fig. 10*A–C*)
ckGnRH	Custom-made (n/a)	Aves	Chicken; monoclonal	1:100	Sections; DAC-647 (Fig. 10*E,F*)
Neurophysin	RN2 (AB_2751000)	A. Robinson	Rabbit; polyclonal	1:12000	Section; DAR-488 (Fig. 8-1*B*)

DAB, 3,3´-diaminobenzidine; DAM, donkey anti-mouse; DAR, donkey anti-rabbit; DAC, donkey anti-chicken.

10.1523/ENEURO.0161-18.2018.f8-1 Figure 8-1 Specificity of OFQ antibody. The specificity of the antibody against OFQ was confirmed using adult and embryonic mouse sections. ***A***, Double immunofluorescence for POMC and OFQ showed that the suprachiasmatic nucleus is a structure containing numerous OFQ fibers, consistent with the regulation of the suprachiasmatic nucleus neurons by OFQ (>Allen et al., 1999). Scale bar, 50 μm. ***B***, The identity of the structure with dense OFQ fibers in ***A*** was confirmed with neurophysin staining (on the consecutive section, a peptide known to be present in the suprachiasmatic nucleus; Sofroniew and Weindl, 1978; van den Pol, 1986). The omission of the primary antibody failed to provide any signal in the same location (the halo on the left is caused by a slight fold in the tissue). Scale bar, 50 μm. ***C***, ***D***, As previously published, OFQ-positive staining was seen in the dorsal root ganglion (***C***; Chen and Sommer, 2006; scale bars: left, 500 μm; right, 50 μm) and cells in the trigeminal ganglion (***D***), where OFQ is known to modulate neurons (Wang et al., 1999; Bongsebandhu-Phubhakdi et al., 2011; same magnification as in ***A***). Download Figure 8-1, TIF file.

### OFQ inhibition in GnRH cells is mediated by G_i/o_-protein-coupled inwardly rectifying potassium channels

**Figure 4.**
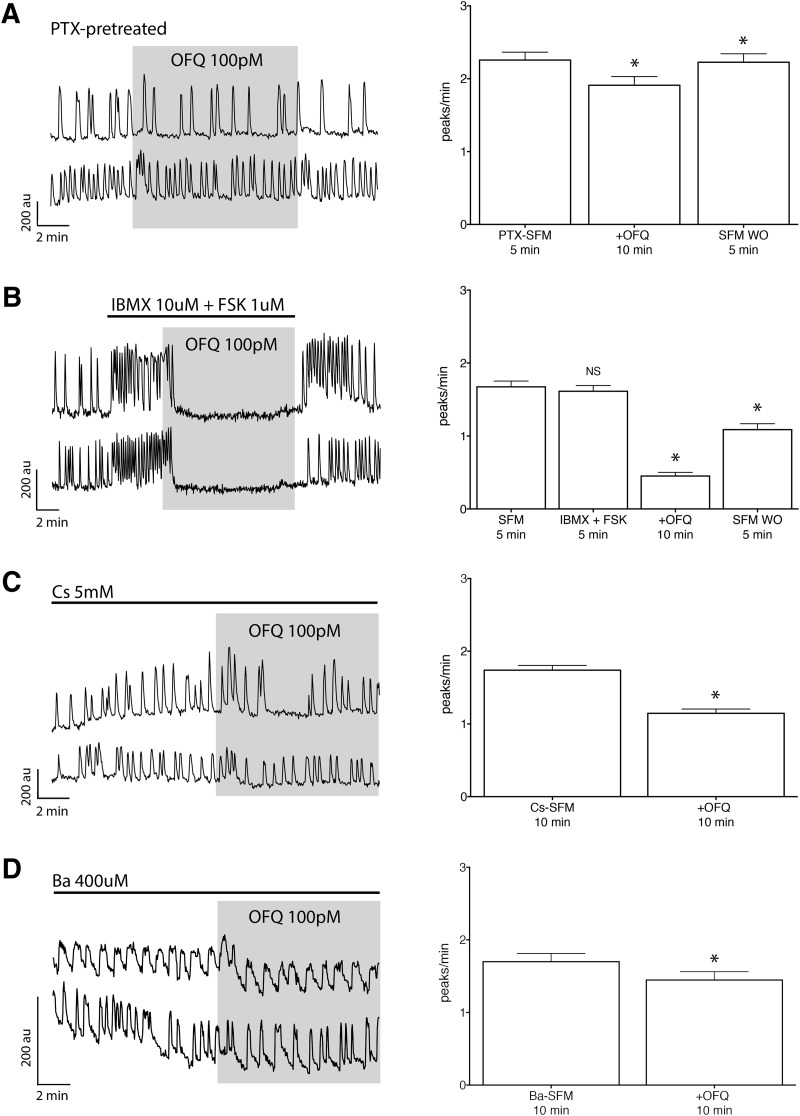
The receptor ORL1 is G_i/o_ coupled and activates GIRK channels to inhibit GnRH neuronal activity. ***A***, Left, Calcium imaging recordings, after a 4 h pretreatment with PTX (250 ng/ml), showing OFQ (100 pm) evoked only a mild inhibition of GnRH neuronal activity. Right, Summary data showing the average frequency of calcium oscillations in all GnRH neurons pretreated with PTX and tested during the control (PTX-SFM), treatment (+OFQ), and washout (SFM WO) periods (*n* = 89, *N* = 3). au, Arbitrary unit. *Statistical significance between two consecutive periods. ***B***, Left, The OFQ-evoked inhibition is insensitive of IBMX and FSK. Right, Summary data showing the average frequency of calcium oscillations in all GnRH neurons tested during control (SFM), pretreatment (IBMX + FSK), treatment (+OFQ), and washout (SFM WO) periods (*n* = 161, *N* = 3). Note that the IBMX + FSK treatment is not showing a significant increase due to the calcium plateau evoked in some cells, which results in an underestimate of peak detection. ***C***, ***D***, Left, The broad-spectrum blocker of K_ir_ channels, Cs or Ba, reduced the OFQ-evoked inhibition. Right, Summary data showing the average frequency of calcium oscillations in all GnRH neurons pretreated with Cs or Ba and tested during the pretreatment (Cs or Ba) and treatment (+OFQ) periods (Cs: *n* = 169, *N* = 5; Ba: *n* = 36, *N* = 4).

It has been reported that ORL1 receptors couple to a G_i/o_-type G-protein (Ikeda et al., 1997). Thus, explants were treated with PTX (250 ng/ml) for >4 h to uncouple G_i/o_-proteins from its receptors. PTX treatment attenuated OFQ inhibition of GnRH neurons (Tables 2, row h, 3, rows a and d, Fig. 4*A*). A canonical pathway downstream of a G_i/o_-type protein is a decrease in the activity of adenylyl cyclase and cyclic adenosine monophosphate. However, coapplication of IBMX (10 µm) and FSK (1 µm), inhibiting phosphodiesterase and activating adenylyl cyclase, respectively, had no effect on OFQ inhibition of GnRH neurons (Tables 2, row i, 3, rows a and e, Fig. 4*B*). Alternatively, G_i/o_-proteins can be coupled to GIRK channels, also known as K_ir_3, voltage-independent potassium channels (Klenke et al., 2010; Constantin and Wray, 2016). The application of OFQ was repeated in the presence of Cs (5 mm) or Ba (400 µm), broad-spectrum blockers of K_ir_ channels, added 10 and 20 min, respectively, before OFQ. Although Cs or Ba did not fully block the OFQ inhibition (Table 2, rows j and k, Fig. 4*C*,*D*; also see Fig. 6*C*), both strongly reduced its magnitude (Table 3, rows a and f or g), supporting the coupling of ORL1 receptors to GIRKs, as previously shown (Wagner et al., 1998).

**Figure 5.**
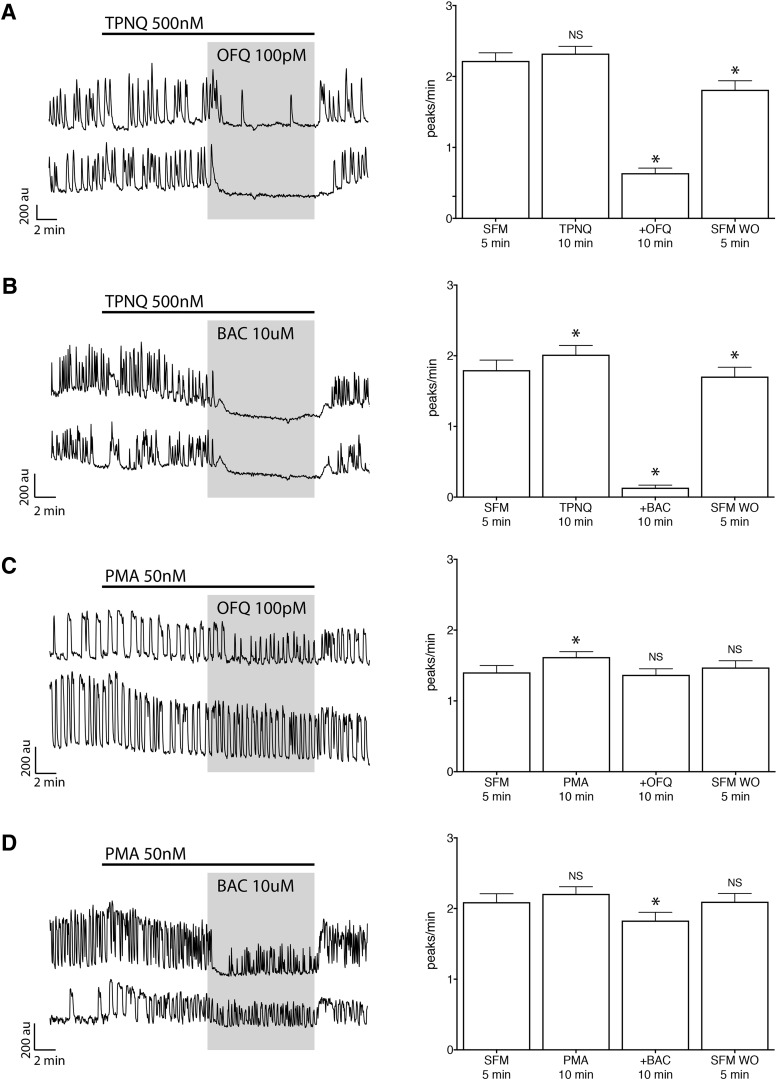
The OFQ-evoked inhibition activates GIRK channels, insensitive to tertiapin-Q but inhibited by protein kinase C phosphorylation. ***A***, Left, Calcium imaging recordings showing that a specific blocker of G-protein inwardly rectifying potassium channels, TPNQ, was ineffective on the OFQ-evoked inhibition. Right, Summary data showing the average frequency of calcium oscillations in all GnRH neurons tested during control (SFM), pretreatment (TPNQ), treatment (+OFQ), and washout (SFM WO) periods (*n* = 80, *N* = 3). au, arbitrary unit. *Statistical significance between two consecutive periods. ***B***, Left, TPNQ was also ineffective on GABA_B_-evoked inhibition with BAC. Right, Summary data showing the average frequency of calcium oscillations in all GnRH neurons tested during control (SFM), pretreatment (TPNQ), treatment (+BAC), and washout (SFM WO) periods (*n* = 20, *N* = 2). ***C***, Left, The protein kinase C activator PMA prevented OFQ-evoked inhibition. Right, Summary data showing the average frequency of calcium oscillations in all GnRH neurons tested during control (SFM), pretreatment (PMA), treatment (+OFQ), and washout (SFM WO) periods (*n* = 118, *N* = 4). ***D***, Left, PMA was also profoundly blunted the GABA_B_-evoked inhibition. Right, Summary data showing the average frequency of calcium oscillations in all GnRH neurons tested during control (SFM), pretreatment (PMA), treatment (+BAC), and washout (SFM WO) periods (*n* = 92, *N* = 3).

Unlike the broad-spectrum K_ir_ blockers cesium and barium, TPNQ is known as a GIRK-specific channel blocker. Unexpectedly, TPNQ (500 nm; Wu et al., 2011), added 10 min before OFQ, did not prevent the OFQ inhibition (Table 2, row l, Fig. 5*A*) and had no effect on its magnitude (Table 3, rows a and h). It is widely agreed that GABA_B_ receptors are mediated by GIRK channels (Kahanovitch et al., 2017), including in GnRH neurons (Zhang et al., 2009). Thus, the effect of TPNQ was tested on the inhibition triggered by the activation of GABA_B_ receptor. As with OFQ, TPNQ had no effect on BAC inhibition (Table 2, rows p and q, Fig. 5*B*) or its magnitude (Table 3, rows j and k), and confirmed the ineffectiveness of TPNQ on GnRH GIRK channels. Naringin (500 µm), which is an activator of GIRK channels using the same binding site as TPNQ (Yow et al., 2011), also did not change GnRH neuronal activity (Table 2, row m).

**Figure 6.**
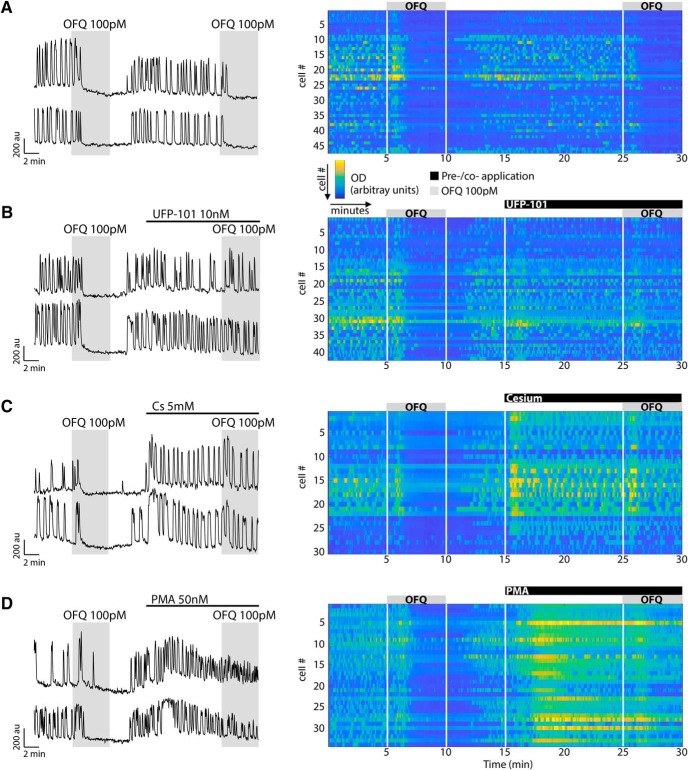
Blockers are effective in GnRH neurons identified as OFQ responsive. ***A–D***, Left, Calcium imaging recordings of explants showing that OFQ (100 pm) evoked a potent decrease in the frequency of calcium oscillations in GnRH neurons (first application, ***A–D***). The inhibition is repeatable (second application, ***A***) and blocked by UFP-101 (ORL1 antagonist), cesium (broad-spectrum blocker of K_ir_ channels), and PMA (PKC activator). ***A–D***, Right, Heatmaps of changes in the levels of intracellular calcium in cells recorded simultaneously during different paradigms (left). Each row represents changes in a single cell. White lines indicate the time of drug application. Note the large number of GnRH cells inhibited by OFQ (first application, ***A–D***), the repeatability of the inhibition (second application, ***A***), and the effectiveness of UFP-101 (ORL1 antagonist), cesium (broad-spectrum blocker of K_ir_ channels), and PMA (PKC activator; ***B***, ***C***, and ***D***, respectively, second application) to prevent OFQ inhibition. Note the homogeneity of the response throughout the cells within an explant.

**Figure 7.**
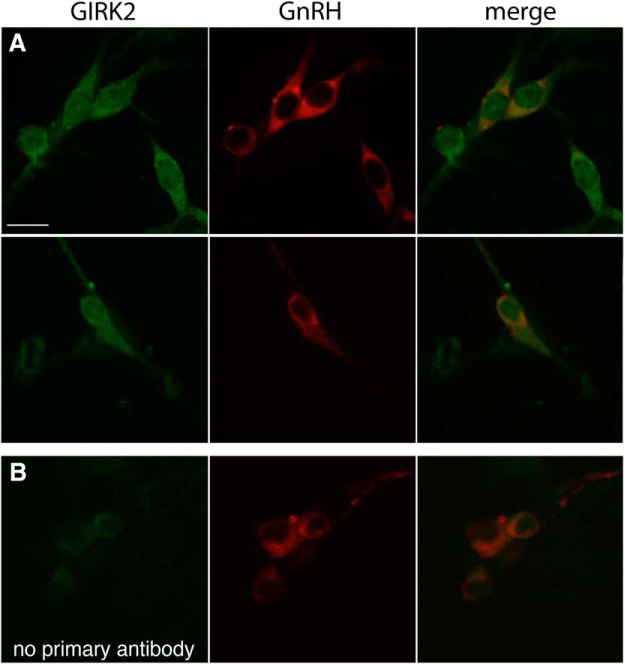
GnRH neurons are immunoreactive for GIRK2 (K_ir_3.2). ***A***, Single confocal plans showing GnRH neurons (red), colabeled with GIRK2 (green; scale bar, 10 μm) in 11-d-old explants (*N* = 3). Representative pictures showing all GnRH neurons examined were colabeled with the GIRK2 antibody. ***B***, Single confocal plans showing GnRH neurons (red), with background (green) when GIRK2 antibody was omitted and used to set the camera at the level of nonspecific staining; the experiment was run simultaneously with the one shown in the second row in ***A***.

GIRK channels are tetramers and can form with three neuronal subtypes, GIRK1 (K_ir_3.1), GIRK2 (K_ir_3.2), and GIRK3 (K_ir_3.3). GIRK2 can form a homotetramer, while GIRK1 and GIRK3 require heteromerization for functionality. Previous RT-PCR analysis had found transcripts for *Kcnj3* (GIRK1) in GnRH neurons (Constantin and Wray, 2016). However, ML297 (10 µm), an activator of GIRK1-containing tetramers (Kaufmann et al., 2013) had no effect on GnRH neuronal activity (Table 2, row n). RT-PCR data also identified transcripts for *Kcnj9* (GIRK3) in GnRH neurons (Klenke et al., 2010). Since GIRK2 and GIRK3 are less sensitive or insensitive to TPNQ, respectively (Nockemann et al., 2013; Whorton and MacKinnon, 2013; Llamosas et al., 2017), an alternative approach was used to clarify the role of GIRK channels in the OFQ inhibition. GIRK channels are inhibited through a PKC-dependent mechanism (Stevens et al., 1999). The PKC activator PMA (50 nm) was applied before OFQ, and OFQ failed to inhibit GnRH neurons (Table 2, row o, Fig. 5*C*; Table 3, rows a and i, Fig. 6*D*). A similar effect was observed with PMA applied before BAC (Tables 2, row r, 3, rows j and l, Fig. 5*D*). These results suggested the absence of GIRK1 but the presence of the other neuronal form, GIRK2, in GnRH neurons. Immunofluorescent staining with a validated antibody (Marron Fernandez de Velasco et al., 2017) demonstrated the presence of GIRK2 in all GnRH neurons (Fig. 7), consistent with its ubiquitous presence in most neurons (Lüscher and Slesinger, 2010). Together, these data are consistent with GIRK2/3 channels, downstream of ORL1 receptor, mediating the OFQ inhibition in GnRH neurons.

### OFQ fibers contact GnRH neurons

**Figure 8.**
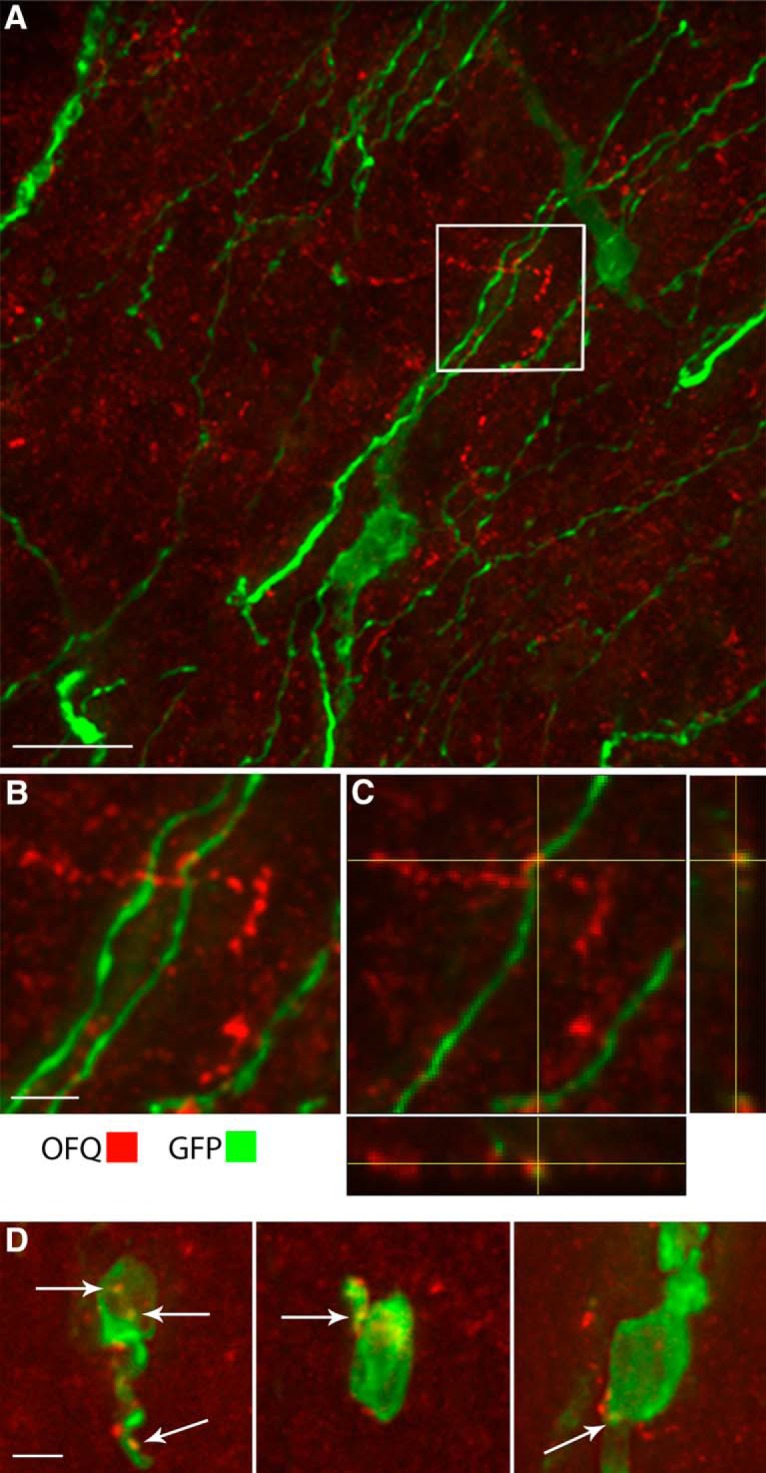
POA GnRH neurons are apposed by OFQ-immunoreactive fibers. ***A***, Representative stack of confocal images showing GnRH neurons labeled with GFP- (green) and OFQ- (red) immunoreactive fibers in the POA of an adult male mouse. Scale bar, 20 µm. Enlarged image from boxed area in ***B***. Scale bar, 5 µm. ***C***, Single confocal plan from the boxed area showing an OFQ-immunoreactive fiber apposed to a GnRH fiber. ***D***, Representative stacks of confocal images showing GnRH neuron cell bodies contacted by an OFQ-immunoreactive fiber (arrows, merged yellow). Scale bar, 5 µm. The specificity of the OFQ antibody is shown in Figure 8-1.

**Figure 9.**
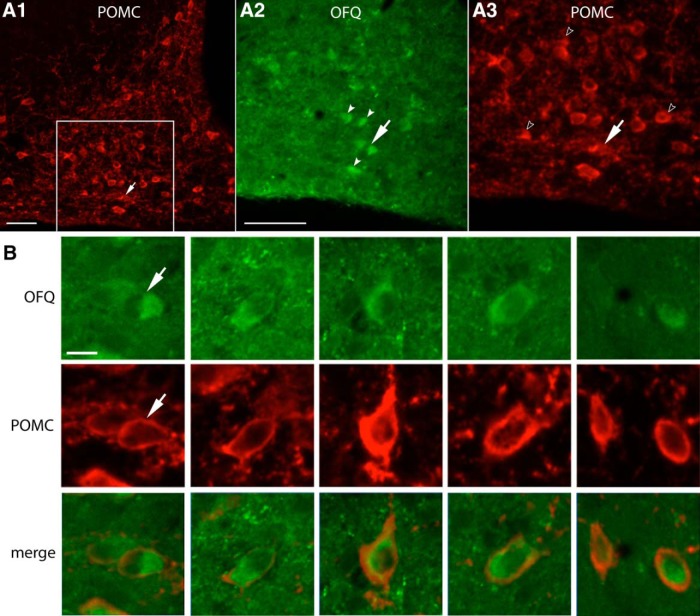
A subset of ARC POMC neurons is colabeled with OFQ. ***A1–A3***, Representative photographs showing ARC neurons immunoreactivity for POMC (red) and OFQ (green) in an adult male mouse. The box in ***A1*** is enlarged in ***A2*** and ***A3*** to show a lateral subset of POMC neurons colabeled with OFQ (low magnification, ***A1***; high magnification, ***A2***, ***A3***). Scale bars, 50 μm. The arrows in ***A2*** and ***A3*** point to the neuron indicated in the high-magnification images in ***B***. White arrowheads in ***A2*** show OFQ neurons colabeled with POMC in ***A3***. Empty arrowheads in ***A3*** show POMC neurons that are not colabeled with OFQ in ***A2***. ***B***, Representative photographs showing examples of ARC POMC neurons coexpressing OFQ. Scale bar, 10 μm.

**Figure 10.**
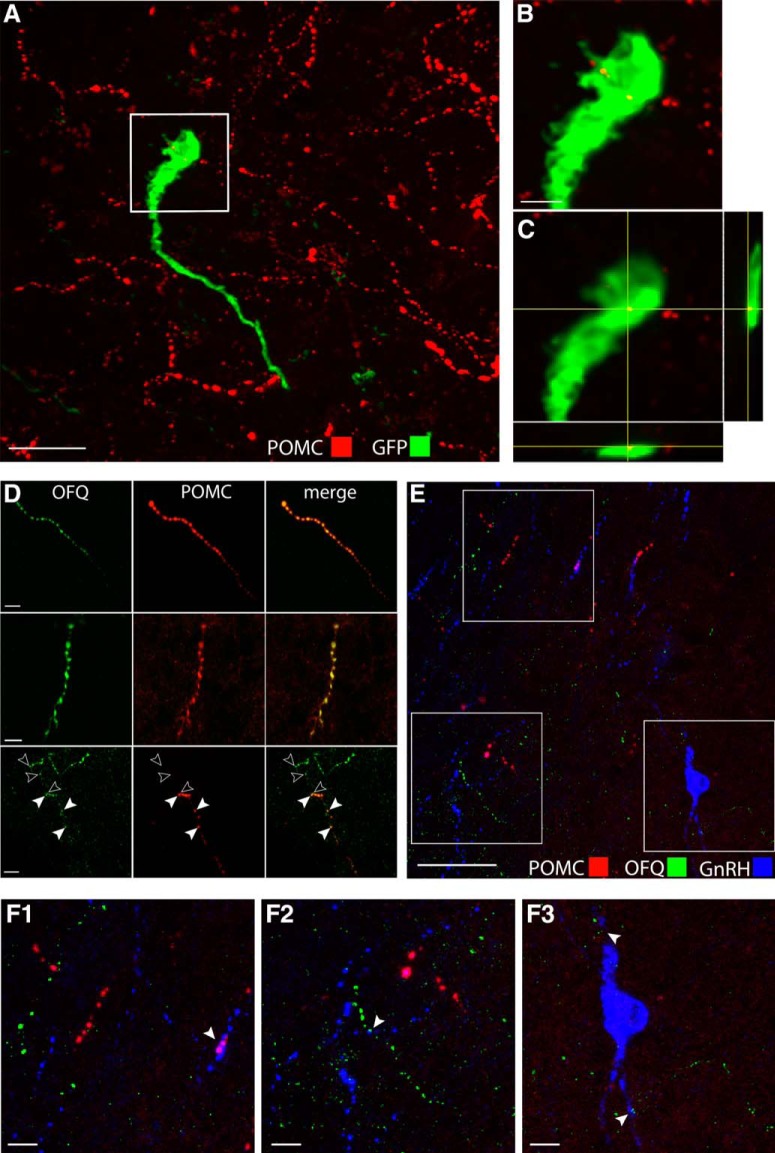
POMC-immunoreactive fibers contact GnRH neurons and POMC fibers in the POA coexpress OFQ. ***A–C***, Representative stack of confocal images showing GnRH neurons labeled with GFP- (green) and POMC- (red) immunoreactive fibers in an adult male mouse. Scale bar, 20 μm. Enlarged image from boxed area in ***B***. Scale bar, 5 μm. ***C***, Single confocal plan from boxed area showing a POMC-immunoreactive fiber apposed to this GnRH cell body in the POA. ***D***, Representative confocal images showing fibers immunoreactive for OFQ and POMC in the POA. Scale bars, 10 μm. Note individual varicosities in the same fiber which are immunolabeled for only OFQ (single-labeled; open arrowheads, third panel) or for both OFQ and POMC (dual-labeled; filled arrowheads), suggesting that spatial localization of these peptides might occur in processes. ***E***, ***F***, Representative confocal image (*Z* projection, ∼10 μm) showing immunoreactivity for OFQ (green) and POMC (red) in fibers surrounding GnRH (blue) neurons in the POA. Scale bar, 50 μm. ***F1–F3***, Boxed areas in ***E*** enlarged show contacts (arrowheads) between GnRH and POMC fibers (***F1***), between GnRH and OFQ fibers (***F2***), and near cell bodies (***F3***). Scale bars, 10 μm.

To begin to identify the anatomic circuit, immunocytochemistry for OFQ and GnRH was performed. In contrast to ewes (Foradori et al., 2007), GnRH neurons in adult mice were not immunoreactive for OFQ. However, OFQ fibers were observed in the POA, where most GnRH cell bodies are found, and within this region some OFQ fibers were found apposed to GnRH fibers and cell bodies (Fig. 8). In the rat, a subpopulation of POMC neurons in the ARC is labeled with OFQ (Maolood and Meister, 2010) and POMC fibers contact GnRH neurons (Leranth et al., 1988; Simonian et al., 1999). Thus, the possibility that OFQ fibers contacting GnRH neurons originated from POMC neurons in the ARC was investigated. In the mouse, a subset (estimated 5–10%) of POMC neurons was immunoreactive for OFQ (Fig. 9), but all OFQ neurons were POMC positive. POMC fibers were observed apposed to GnRH neurons in the POA (Fig. 10*A–C*). Notably, the POA also contained some fibers colabeled with OFQ and POMC (Fig. 10*D*). However, using triple immunolabeling for GnRH, OFQ, and POMC, fibers colabeled with POMC and OFQ were not found contacting GnRH neurons (Fig. 10*E*,*F*).

### OFQ inhibits GnRH neurons *in situ*, independently of GABAergic and glutamatergic inputs

**Figure 11.**
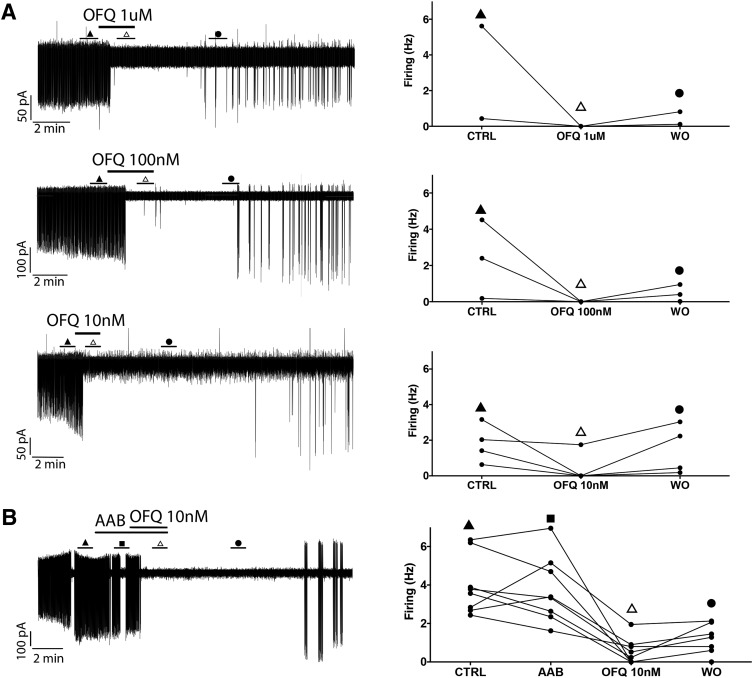
OFQ inhibits the GnRH neuron firing rate in acute brain slices, independent of GABAergic and glutamatergic inputs. ***A***, Left, Electrophysiological recording of adult GFP-tagged GnRH neurons showing that OFQ (1 µm, 100 nm, and 10 nm) evoked a potent decrease in GnRH neuron firing rate. Right, Summary data showing quantification of firing rate (in Hz) in individual GnRH neurons tested at the different concentrations during the control (CTRL), treatment (OFQ), and washout (WO) periods. The values represent the average of 1 s bins for the last 1 min of each period identified on the traces. ***B***, Left, OFQ (10 nm) in the presence of AABs (BIC, 20 µm; CNQX, 10 µm; d-AP5, 20 µm) still decreased GnRH neuron firing rate. Right, Summary data showing quantification of the firing rate (in Hz) in individual GnRH neurons tested during the control (CTRL), pretreatment (AAB), treatment (+OFQ), and washout (WO) periods. The values represent the average of 1 s bins for the last 1 min of each period identified on the traces.

**Figure 12.**
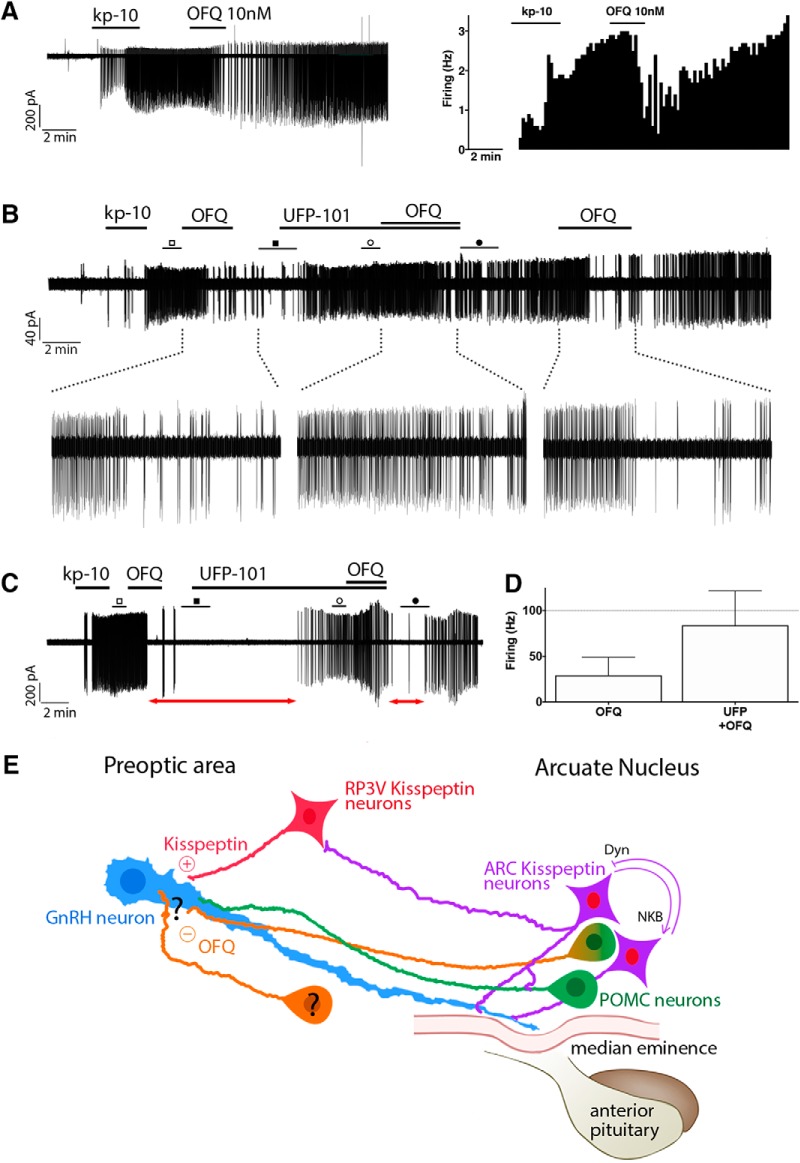
OFQ as a new player in the ARC regulation of GnRH neurons. ***A***, Left, Electrophysiological recording of an adult GFP-tagged GnRH neuron showing OFQ (10 nm) suppressed the firing rate of kisspeptin-10 (kp-10; 100 nm)-activated GnRH neurons. Right, Corresponding periodogram illustrating the changes in GnRH neuron firing rate (in Hz) over time (i.e., the increase in firing rate induced by kp-10 and its suppression by OFQ). The bars represent the instant frequency in 10 s bins. ***B***, Electrophysiological recording of another adult GFP-tagged GnRH neuron showing OFQ (10 nm) suppressed the firing rate of kp-10 (100 nm)-activated GnRH neurons. The inhibition evoked by OFQ was partially antagonized by UFP-101 (500 nm) but was reestablished by a second application of OFQ after the washout of UFP-101. ***C***, Electrophysiological recording of a third adult GFP-tagged GnRH neuron showing the partial antagonism of OFQ by UFP-101. Note that, without UFP-101, the OFQ evoked a pause in the tonic firing for ∼11 min (red arrow, on the left), while with UFP-101 the pause was ∼2.5 min (red arrow, on the right; i.e., an approximately four times faster recovery despite being further from the initial kp-10 application. ***D***, Quantification of the OFQ inhibition, before (square) and after (circle) the application of UPF-101. The values represent the firing rate 4 min after OFQ or UFP-101 plus OFQ application, normalized to the firing rate in the last minute of aCSF application before application of OFQ (empty square) or UFP-101 before UFP-101 plus OFQ (empty circle) application, respectively. ***E***, Diagram representing where OFQ stands within the already established connectivity around GnRH neurons. GnRH neuron (blue) receive excitatory inputs from RP3V kisspeptin neurons (red) at the soma. ARC kisspeptin neurons (purple), autoregulated via a neurokinin B and dynorphin loop, contact GnRH neuronal processes around the median eminence. Both kisspeptin neuronal populations are linked via ARC → RP3V connections. POMC neurons (green) contact GnRH neurons (Leranth et al., 1988; Simonian et al., 1999). ARC kisspeptin neurons contact POMC neurons (Fu and van den Pol, 2010; Nestor et al., 2016). OFQ fibers (orange), possibly originating from a subpopulation of POMC neurons and/or another unidentified neuronal population, contact GnRH neurons, providing an inhibitory signal.

OFQ was applied at different doses to GnRH neurons in acute brain slices. OFQ stopped the firing in two of two cells (*N* = 2), three of three cells (*N* = 2), and two of two cells (*N* = 1) at 1 µm, 100 nm, and 10 nm, respectively (Fig. 11*A*). After the blockade of GABAergic and glutamatergic excitatory inputs with AAB (BIC, 20 µm; CNQX, 10 µm; d-AP5, 20 µm), OFQ (10 nm) still inhibited eight of nine cells, five of them being stopped (*N* = 3; Fig. 11*B*). Of the 12 cells that stopped, without (*n* = 7) or with AAB (*n* = 5), the average time to regained firing was after 9.0 ± 1.3 min (*N* = 8). OFQ (10 nm) suppressed kisspeptin-10 (100 nm)-evoked excitation (Fig. 12*A–C*; four of four cells tested; *N* = 4) but did not terminate it. The OFQ suppression was partially prevented by UFP-101 (500 nm; *N* = 3), supporting the role of ORL1 (Fig. 12*B–D*). These data support a role for OFQ as a modulator of GnRH neuron neuronal activity but rule out its function as the terminator of kisspeptin-induced excitation.

## Discussion

The present study investigated the neuromodulation of GnRH neurons by OFQ in the mouse. We report that OFQ strongly inhibits spontaneous GnRH neuronal activity and can repress kisspeptin-evoked excitation. The inhibition, independent of GABAergic and glutamatergic inputs, is mainly mediated by ORL1, G_i/o_-protein-coupled receptor, and the subsequent activation of GIRK channels. *In vivo*, OFQ-immunopositive fibers were found contacting GnRH neurons. Together, these data suggest that OFQ can downregulate the reproductive axis.

OFQ inhibits GnRH secretion from mediobasal fragments (Dhandapani and Brann, 2002; An et al., 2005) or *in vivo* [push–pull (An et al., 2005); intracerebroventricular (An et al., 2005, 2009)]. The *in vivo* inhibition is ORL1 dependent (An et al., 2005, 2009). However, since the receptor is widely distributed in the brain (Ikeda et al., 1998; Neal et al., 1999; Houtani et al., 2000) and even in the hypothalamus (Chen et al., 2017), how OFQ inhibited GnRH secretion was unknown. Evidence for indirect actions of OFQ on GnRH neurons exist, including the following: (1) transcripts for ORL1 were not detected in an immortalized GnRH cell line obtained from mouse (Dhandapani and Brann, 2002); and (2) the stimulatory effect of ORL1 antagonists on POA GnRH release were mediated by glutamate in male rats (An et al., 2008). However, our calcium imaging and patch-clamp data indicate a direct action of OFQ onto GnRH neurons since OFQ inhibited GnRH neuronal activity while both GABAergic and glutamatergic inputs were disrupted. Notably, GnRH neuronal activity does not require these two excitatory inputs (Constantin et al., 2010; Lee et al., 2012), but they are the main excitatory inputs in acute brain slices (Iremonger et al., 2010; Herbison and Moenter, 2011) and largely contribute to GnRH neuronal activity in nasal explants (Constantin et al., 2010). Inhibition of GnRH neuronal activity by OFQ is consistent with previous data showing OFQ hyperpolarized GnRH neurons from OVX guinea pig through an inwardly rectifying potassium current (Wagner et al., 1998). A direct action of OFQ on GnRH neurons is further supported by our findings of ORL1 transcript in GnRH neurons and OFQ fibers contacting GnRH neurons.

Examination of the signaling pathway demonstrated that the OFQ inhibition was ORL1 mediated. ORL1 coupling to G_i/o_-type G-protein (Reinscheid et al., 1995; Ikeda et al., 1997) was confirmed in GnRH neurons treated with PTX. OFQ-induced inhibition could occur through two signaling pathways: a decrease of cyclic adenosine monophosphate and/or the activation of GIRK channels (for review, see Al-Hasani and Bruchas, 2011). The data obtained using IBMX and FSK clearly indicate that the OFQ-induced inhibition of GnRH neuron activity occurs without a decrease of cAMP. In fact, previous studies from our laboratory have already shown that a decrease of cAMP does not affect GnRH neuronal activity (Constantin and Wray, 2008, 2016), while the activation of Cs- and Ba-sensitive channels inhibits GnRH neurons (Klenke et al., 2010; Constantin and Wray, 2016).

Most neurons express GIRK1, GIRK2, and GIRK3 (Lüscher and Slesinger, 2010). Transcripts for both GIRK1 (K_ir_3.1, *Kcnj3*; Constantin and Wray, 2016) and GIRK3 (K_ir_3.3, *Kcnj9*; Klenke et al., 2010) subunits are expressed in GnRH neurons. Neither GIRK1 nor GIRK3 subunits can form homotetrameric functional GIRK channels but can assemble into functional heterotetramers (Lüscher and Slesinger, 2010). The sensitivity of the OFQ inhibition to Cs and Ba, but not to TPNQ, naringin, or ML297, indicated a different subunit composition of the GIRK channels associated with ORL1 (Kobayashi et al., 2011) in GnRH cells and suggested the presence of GIRK2 subunits in their GIRK channels. To further test this hypothesis, baclofen, a GABA_B_ agonist, was used to activate GIRK channels (Kahanovitch et al., 2017). Although GABA_B_ can use two-pore domain potassium channels (Bushell et al., 2002; Deng et al., 2009), the literature supports the role of GABA_B_ using inwardly rectifying potassium currents in GnRH neurons (Wagner et al., 1998; Zhang et al., 2009). BAC-induced inhibition was also insensitive to TPNQ. This observation is supported by a study showing that GIRK channels coupled to ORL1, at least partially, overlap the GABA_B_-coupled GIRK channel pool (Wagner et al., 1998). Since no other GIRK-specific blockers are available, an alternative route to test GIRK2 involvement in OFQ signaling via ORL1 was used. Protein kinase C is known to phosphorylate GIRK channels, GIRK2 included (Adney et al., 2015), and to desensitize GIRK-mediated currents (Stevens et al., 1999; Lüscher and Slesinger, 2010). Together with our earlier data, the lack of OFQ inhibition after the application of a PKC activator and the immunoreactivity of GnRH neurons for GIRK2 show that OFQ inhibition of GnRH neurons is mediated by GIRK channels containing GIRK2/3 subunits.

OFQ fibers were identified contacting GnRH fibers and cell bodies in the POA, supporting a direct effect of OFQ on GnRH cells. One could argue that the contacts on GnRH fibers are irrelevant to the modulation of GnRH neuron firing; however, GIRK channels localize to specific subcellular compartments (Kulik et al., 2006). GIRK2 splice variants exhibit different subcellular distribution and might impact the integration of afferent inhibitory inputs (Marron Fernandez de Velasco et al., 2017). In addition, GABA_B_ inhibition in GnRH neurons is largely lost in preparations that sever processes (Constantin et al., 2012). GnRH neuronal cell bodies were not immunolabeled for OFQ in the adult mouse. This is in contrast to data from the sheep where virtually all GnRH neurons express OFQ in ewes (Foradori et al., 2007). GnRH neuron immunoreactivity for OFQ in sheep might be a species-specific and lasting vestige of their embryonic origin. Indeed, the literature shows that OFQ is highly expressed in early development (Ikeda et al., 1998; Neal et al., 2001) and is involved in the olfactory placode formation (Lleras-Forero et al., 2013) where GnRH cells originate (Schwanzel-Fukuda and Pfaff, 1989; Wray et al., 1989a). Alternatively, the active transport of OFQ down the axon could have precluded the identification of OFQ-labeled GnRH neuronal cell bodies in the mouse. However, two facts seem to argue against this: (1) the median eminence, which contains a high density of GnRH neuronal fibers, was not labeled for OFQ; and (2) cell bodies immunopositive for OFQ were present in the ARC, in agreement with data from rat and sheep (Maolood and Meister, 2010; Nestor et al., 2013).

In the current model of GnRH pulsatility, ARC kisspeptin neurons exhibit autonomous rhythmicity, driven by neurokinin B and autocrine inhibition by dynorphin A, and lead to the excitation of GnRH neurons (Mittelman-Smith et al., 2012; Navarro, 2012; Goodman et al., 2013). Based on the long-lasting response to kisspeptin that has been recorded in GnRH neurons after exogenous application of kisspeptin-10 (Han et al., 2005; Constantin et al., 2009, 2012) or electrical stimulation of AVPV fibers contacting GnRH neurons (i.e., evoked release of endogenous kisspeptin; Liu et al., 2011), it is reasonable to assume that GnRH neurons do not release an autocrine inhibitor and, therefore, require a third partner to return their electrical activity to baseline. The cell type/neuropeptide required for this is unknown. The ARC is central to negative feedback (Xu et al., 2011; Yeo and Herbison, 2014), and POMC neurons expressing ERα and progesterone receptor have been implicated in this process (Lagrange et al., 1995; Xu et al., 2011; Nestor et al., 2013); however, the mechanism remains unknown. The presence of OFQ neurons in the ARC whose expression is upregulated by estradiol (Sanathara et al., 2014), combined with the ability of OFQ to directly suppress GnRH excitation triggered by kisspeptin-10, points to ARC cells expressing OFQ that communicate to GnRH cells as a viable candidate. The *POMC* gene belongs to the opioid/orphanin gene family (Navarro et al., 2016) and POMC and OFQ share processing enzymes (Allen et al., 2001). As such, the coexpression of OFQ and POMC already described in the rat and sheep (Maolood and Meister, 2010; Nestor et al., 2013), was now observed in the mouse. Despite the presence of OFQ and POMC fibers in the POA and the identification of colabeled fibers in this region, colabeled fibers were not detected in contact with GnRH neurons. Thus, afferents from another OFQ-positive neuronal population cannot be excluded (Fig. 12*E*). However, one explanation for not detecting colabeled fibers in contact with GnRH neurons might be the paucity of the OFQ/POMC contacts associated with the cell sampling. Another explanation might be differential routing of the neuropeptides into different processes, as seen in vasopressin neurons (Landry et al., 2003) and neurons expressing RFamide-related peptides (Yano et al., 2003). Thus, the exact identity of the source of OPQ fibers contacting GnRH neurons warrants further investigation.

In summary, using calcium imaging, immunohistochemistry, and electrophysiology, we have confirmed that OFQ can inhibit GnRH neurons and suppress, but not end, kisspeptin activation of GnRH neurons. Together, these results bring into light a potent neuromodulator of GnRH neurons in the mouse and contribute to our knowledge of the neuronal network upstream of GnRH neurons.

## Synthesis

Reviewing Editor: Jeffrey Blaustein, University of Massachusetts Decisions are customarily a result of the Reviewing Editor and the peer reviewers coming together and discussing their recommendations until a consensus is reached. When revisions are invited, a fact-based synthesis statement explaining their decision and outlining what is needed to prepare a revision will be listed below. The following reviewer(s) agreed to reveal their identity: Michael Lehman, Suzanne Moenter.

Unfortunately, the reviewers have identified a number of remaining issues that need to be addressed before this paper is acceptable for publication.

Discussion, lines 518-524: The authors suggest that the lack of colocalized POMC/OFQ fibers in contact with GnRH neurons is due to the differential routing of peptides. As support for this, they note that POMC/OFQ fibers were seen in the POA, that then appeared labeled for only OFQ (Fig. 10D). However, in the indicated panel of this figure (bottom row of panel D), it is impossible to definitively tell whether this is a single fiber, or a double-labeled fiber intertwined with a single-labeled OFQ fiber (confocal analysis of a Z stack would be needed to determine this). It certainly is possible that OFQ and POMC could be sequestered in different vesicles and fiber varicosities, but from the images shown in Fig. 10, it really looks like they are almost completely co-localized in those fibers which are double-labeled.

In addition, the fact that the SCN is innervated by the ARC, and that the SCN also contains OFQ but not POMC, is not strong evidence that OFQ fibers in the SCN arise from the ARC. Indeed, I don't believe there is compelling evidence for POMC innervation of the SCN in any species. Thus, the second half of the statement (lines 522-524) is also not supportive of the differential routing of OFQ and POMC from a single set of ARC neurons. Instead, the author should note the possibility that POMC/OFQ neurons may not be the physiological mediators of OFQ effects on GnRH neurons, and that other OFQ neurons may instead be involved. Alternatively, POMC/OFQ neurons could act indirectly to influence GnRH neuronal activity, even perhaps via other OFQ neurons.

While absence of proof is not proof of absence, the weight of the data in this study suggests that the majority of OFQ fibers innervating GnRH neurons do not originate in the POMC/OFQ subset in the ARC. Thus, statements such as “these fibers appear to originate from a subpopulation of POMC ARC neurons” (lines 21-22), and “we demonstrate that the OFQ fibers contacting GnRH neurons in the POA likely originated in POMC neurons in the ARC” (lines 92-94), should be significantly toned down and amended to more accurately reflect the unresolved origin of this input, and that OFQ fibers contacts onto GnRH neurons may just as likely arise from other sets of OFQ neurons besides the ARC.

There remain concerns about overinterpretation both of this work and of the literature with regard to what can be concluded from these experiments:

The duration of GnRH release relative to kisspeptin application can be determined from Glanowska but not relative to kisspeptin signaling, for example (line 51). In the discussion, the work of Wagner and Liu (463-4) can be used to identify that a potassium channel is likely involved but not which one as the measure used to suggest potassium channel (reversal potential) would be the same for all of them.

Fig 2. The authors state that no apparent difference is attributable to sex but the data are confusing. Line 307 states 14 explants, but the legend and tables indicate 5 and fewer at individual doses. This may be insufficient to know if there is a sex difference. Possible lack of sufficient power should be addressed.

The low explant n (1 or 2 in some cases) is a problem. The authors adjust the P value for significance, but the use of individual cells to such an extent as biological replicates may require a more rigorous correction. There are a couple hundred cells in some (the statistically used n) and only 5-6 explants; in another example n of 1 explant has 66 cells. This should be dealt with in the statistical analysis.

The data in Fig 12 are too sparse and inconsistent to make a conclusion. The text (L408) says OFQ inhibition was prevented by UPF but the legend (and the data) suggests this was partial. In part C the kisspeptin response appears to have terminated before the second application of OFQ. The paper would be stronger without trying to overconclude on a point that the data are too sparse to support.

There are also some minor issues that need to be fixed:

Please identify Fig 7 as section vs explant

311-312 needs to be rewritten as autonomous means independent of regulation.

314 Alreja has published a postsynaptic effect of mGluRs in GnRH neurons and should be cited as a counterpoint to Chu and Moenter.

415 More correct to say “mediated (at least) in part” given inhibition of these receptors reduced but did not eliminate response.

416-417 needs to be rewritten as it implies GnRH neurons come from a subpopulation of POMC neurons

459 -fragment

497 Liu et al did not measure kisspeptin release but the wording here makes it seem as if they did.
