# Supplementary figures and images for: Nociceptin/Orphanin-FQ Inhibits Gonadotropin-Releasing Hormone Neurons via G-Protein-Gated Inwardly Rectifying Potassium Channels
Source: eNeuro. 2018 Dec 26;5(6):ENEURO.0161-18.2018. doi: 10.1523/ENEURO.0161-18.2018 (PMC6325553; doi:10.1523/ENEURO.0161-18.2018)

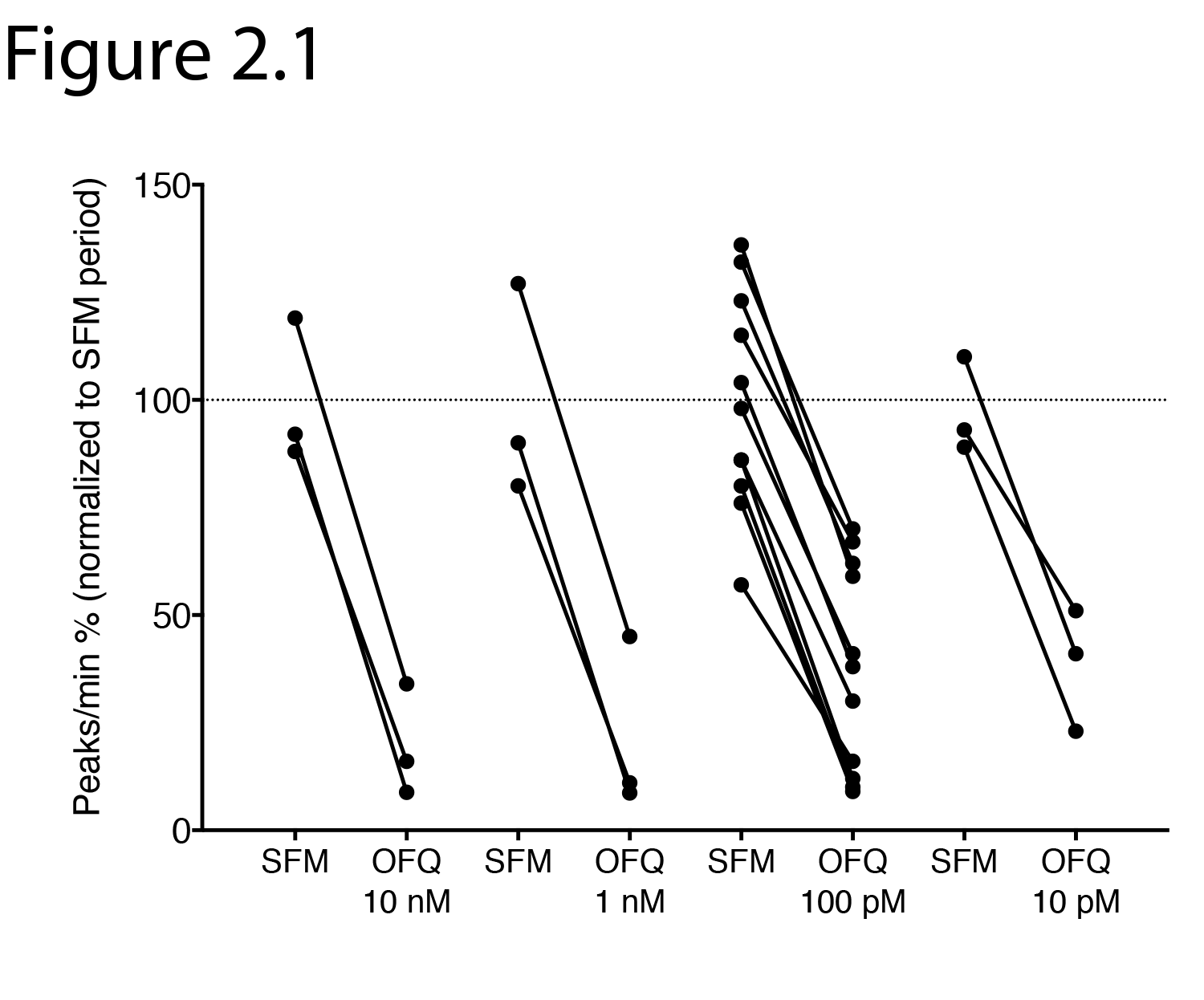

Supplement: Figure 2-1 — The response to OFQ is consistent throughout the explants. The graph represents the average inhibition calculated per individual explant (N = 14) at the four OFQ doses tested. All explants (unsexed) displayed a similar level of inhibition, ruling out the influence of gender in the OFQ inhibition. Download Figure 2-1, TIF file. [file sup_enu-eN-NWR-0161-18-s01.tif]

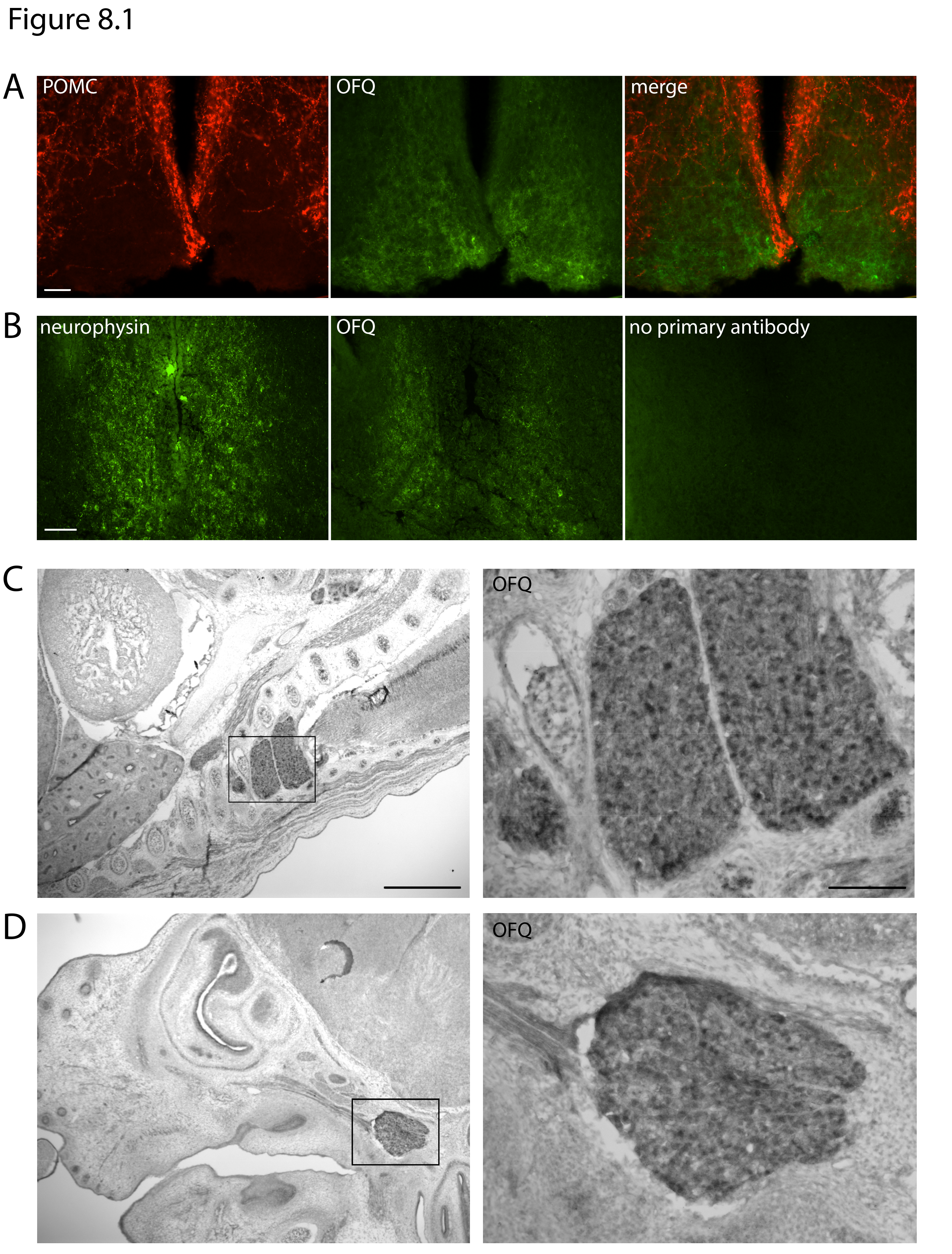

Supplement: Figure 8-1 — Specificity of OFQ antibody. The specificity of the antibody against OFQ was confirmed using adult and embryonic mouse sections. A, Double immunofluorescence for POMC and OFQ showed that the suprachiasmatic nucleus is a structure containing numerous OFQ fibers, consistent with the regulation of the suprachiasmatic nucleus neurons by OFQ (>Allen et al., 1999). Scale bar, 50 μm. B, The identity of the structure with dense OFQ fibers in A was confirmed with neurophysin staining (on the consecutive section, a peptide known to be present in the suprachiasmatic nucleus; Sofroniew and Weindl, 1978; van den Pol, 1986). The omission of the primary antibody failed to provide any signal in the same location (the halo on the left is caused by a slight fold in the tissue). Scale bar, 50 μm. C, D, As previously published, OFQ-positive staining was seen in the dorsal root ganglion (C; Chen and Sommer, 2006; scale bars: left, 500 μm; right, 50 μm) and cells in the trigeminal ganglion (D), where OFQ is known to modulate neurons (Wang et al., 1999; Bongsebandhu-Phubhakdi et al., 2011; same magnification as in A). Download Figure 8-1, TIF file. [file sup_enu-eN-NWR-0161-18-s02.tif]
